# Combining single-cell and bulk RNA sequencing, NK cell marker genes reveal a prognostic and immune status in pancreatic ductal adenocarcinoma

**DOI:** 10.1038/s41598-024-65917-1

**Published:** 2024-07-01

**Authors:** Yonghao Ouyang, Rongxi Shen, Lihua Chu, Chengchao Fu, Wang Hu, Haoxuan Huang, Zhicheng Zhang, Ming Jiang, Xin Chen

**Affiliations:** 1 Research Institute of General Surgery, Jinling Hospital, Nanjing University Medical School, 305 Zhong Shan East Road, Nanjing, 210002 China; 2 Nanchang University, 461 Bayi Avenue, Nanchang, 330006 Jiangxi China; 3 Jinggangshan University, Ji’an, 334000 China; 4 Jiangxi University of Chinese Medicine, Nanchang, 330000 China

**Keywords:** Pancreatic ductal adenocarcinoma, Single-cell RNA-sequencing, NK cell marker genes signature, Prognosis, Immunotherapy, Cancer, Immunology, Biomarkers, Diseases, Medical research, Molecular medicine, Oncology, Risk factors

## Abstract

The NK cell is an important component of the tumor microenvironment of pancreatic ductal adenocarcinoma (PDAC), also plays a significant role in PDAC development. This study aimed to explore the relationship between NK cell marker genes and prognosis, immune response of PDAC patients. By scRNA-seq data, we found the proportion of NK cells were significantly downregulated in PDAC and 373 NK cell marker genes were screened out. By TCGA database, we enrolled 7 NK cell marker genes to construct the signature for predicting prognosis in PDAC patients. Cox analysis identified the signature as an independent factor for pancreatic cancer. Subsequently, the predictive power of signature was validated by 6 GEO datasets and had an excellent evaluation. Our analysis of relationship between the signature and patients’ immune status revealed that the signature has a strong correlation with immunocyte infiltration, inflammatory reaction, immune checkpoint inhibitors (ICIs) response. The NK cell marker genes are closely related to the prognosis and immune capacity of PDAC patients, and they have potential value as a therapeutic target.

## Introduction

Pancreatic cancer is one of the most malignant tumors in the world, with an average 5-year survival rate of only about 5–10%^1,2^. PDAC is the most common pathological type of pancreatic cancer, and as a leading cause of cancer deaths worldwide, its global burden has approximately doubled in the past 25 years^3,4^. Radical surgery is currently the only possible cure for early-stage PDAC^5^. However, because of the insidious onset of PDAC and the early non-specific symptoms, most PDAC patients are already in the inoperable stage when diagnosed^4^. In the past few decades, with the progress in surgery, chemotherapy, etc., the prognosis of PDAC patients has improved dramatically, but the 5-year survival rate remains low^6^. Therefore, it is urgent to search for a treatment for PDAC.

In recent years, as PDAC therapies have been intensively studied, immunotherapy has emerged as the fourth tumor treatment technique after surgery, radiotherapy, and chemotherapy^7^. Immunotherapy has shown unique advantages and shown good efficacy in the treatment of a variety of cancers (e. g., melanoma, renal cell cancer, prostate cancer, urothelial cancer, and ovarian cancer, etc.)^8^. At present, PDAC immunotherapies (mainly including immune checkpoint blockage therapy, adoptive cell transfer and cancer vaccines, either alone or in combination with chemoradiotherapy and other targeted therapies) are still in clinical trials, but effectiveness of these therapies is not satisfactory^9^. Most clinical studies indicate that the effect of any immunotherapeutic agent for PDAC is limited, which is determined by the suppressive immune microenvironment specific to PDAC^10^.

Numerous reports have shown that TME plays a key role in the occurrence and development of PDAC, which in turn affects the prognosis and immunotherapy response of PDAC patients^11^. The TME of PDAC, characterized myeloid-derived suppressor cells (MDSCs) infiltration and poor T cell infiltration, also limits the effectiveness of its immunotherapy^12^. As an important component of TME, past studies have found that NK cell level and activity are significantly downregulated in PDAC, and the methods that promote NK cell function and increase NK cell infiltration in PDAC can effectively improve the prognosis of PDAC patients^13–15^. These imply that NK cells may play an essential role in immunity against PDAC. NK cells belong to innate immune cells, which are able to identify and directly target the cytotoxic effects on primary and metastatic tumor cells through the primary cytotoxic receptors CD16, NKG2D, DNNAM-1 and NCRs, etc.^16^. In addition, NK cells can have paracrine effects with anti-tumor immune cells such as dendritic cells, macrophages, T cells, and endothelial cells through the binding of chemokines and cytokines^17^. Additional reports indicate that activated pancreatic stellate cells (PSC) which constitute the PDAC dense connective tissue and immunosuppressive matrix cancer associated fibroblast (CAF) subset can be targeted by NK cells through NKG2D-MICA/B, and mediate the lysis of PSC^18^. Based on the importance of NK cells in anti-PDAC, there is a need to explore the expression profile and the ability of immunotherapy prediction of NK cells.

“The fundamental operative unit of a cancer is the genetically and epigenetically innovative single cell”^19^. The scRNA-seq technique has been exploited in the TME of various cancers and has enabled comprehensive studies of the transcriptomes of individual cell populations^20^. Identification of prognostic biomarkers to determine therapeutic efficacy and predict prognosis is important for tumor Individualized immunotherapy^21^. The marker genes of a certain type cell has expression characteristics is unique to other kinds of genes, which to some extent can represent the traits of this type cell. In our study, the scRNA-seq data and the bulk RNA-seq data of PDAC were combined to identify NK cell marker genes. Subsequently, NK cell marker genes signature (NKCMGS) was established to predict outcomes and performed the validation of PDAC. Moreover, we also evaluated the relationship between NKCMGS and PDAC immunity by various methods.

## Materials and methods

### Data collection

The scRNA-seq data were downloaded from the GSE212966 dataset of the GEO database (https://www.ncbi.nlm.nih.gov/geo/). After excluding incomplete and file damaged data (3 pancreatic adjacent samples), corresponding 3 pancreatic adjacent samples and 6 pancreatic cancer tissue samples were included, and these data were used to identify NK cell marker genes. A total of 176 PAAD-TCGA bulk RNA-seq and clinical data were downloaded from the UCSC Xena database (http://xena.ucsc.edu/) to establish the NKCMGS. To verify the prognostic prediction power of NKCMGS, we included 6 GSE datasets from the GEO database, including GSE21501 (N = 102), GSE28735 (N = 43), GSE57495 (N = 63), GSE62452 (N = 66), GSE71729 (N = 125) and GSE78229 (N = 49). These datasets retained only the tumor samples and excluded samples with missing data on survival information (including survival time and vital status).

### Identification of the NK cell marker genes and NK cell proportion

By “Seurat” and “SingleR” packages of R language (version 4.1.1), we completed these processes. We removed Low-quality cells with mitochondrial genes greater than 5% or the number of identified genes less than 50. The top 1500 representative genes (which fluctuated greatly across cells) were used in the principal component analysis (PCA). The top 20 principal components (PC, P < 0.05) were subjected for T-distributed stochastic neighbor embedding (t-SNE) clustering. We used genes with “P value < 0.01 and | log2 (fold change) | > 1” as the marker gene of each cell cluster.

### Establishment and verification of NKCMGS

Cox regression, least absolute contraction, and selection operator (Lasso) regression were performed using the “survival”, “glmnet”, “survivalROC”, and “survminer” packages in the R language (version 4.1.1), and area under the receiver operating characteristic curve (ROC), are under curve(AUC) and Kaplan–Meier curve were plotted. The NK cell marker genes associated with overall survival (OS) in PDAC patients were selected by univariate Cox regression (criteria: P < 0.05). With the help of Lasso regression, we selected representative genes from the OS associated NK cell marker genes (above the minimum standard). Stepwise multivariate Cox regression optimized the prognostic model, and calculate the risk score were determined based on the regression coefficients. Based on the median risk score, we divided patients into high-risk and low-risk groups. ROC curves were used to evaluate the prognosis predictive ability of risk scores. By Kaplan–Meier curve, we evaluated survival in different groups. Subsequent validation was performed in six GSE datasets.

### Enrichment analysis

“clusterProfiler”, “org.Hs.eg.db”, ”enrichplot” and “ggplot2” packages of R (version 4.1.1) were used to perform GO and KEGG enrichment analysis^22–24^.

### Immune infiltration analysis

The infiltration of immune cells in the 22 PDAC patients in TCGA was assessed through the CIBERSORT website (https://cibersortx.stanford.edu/)^25^. With the LM22 reference data set, 22 different immune cell infiltrates were analyzed by R language (version 4.1.1).

### T/B cell receptor (T/BCR) diversity analysis and enrichment analysis

By the Shannon index and Richness index of TCR (BCR) of PDAC patients in TCGA provided by Thorsson et al., we analyzed and compared the diversity (Shannon score) and enrichment (enrichment score) of TCR (BCR) in different risk groups^26^.

### Evaluation of inflammatory response

“GSVA” package of R language was performed to calculate score of 7 metagenes which represent different inflammation^27^. Subsequently, the association between NKCMGS and metagenes was evaluated by spearman correlation analysis.

### Evaluation of immunotherapy response

The response to immunotherapy in the high-risk and low-risk groups were evaluated by PD-L1 protein expression, tumor mutational burden (TMB), neoantigen, Tumor immune dysfunction and exclusion (TIDE). The PD-L1 protein expression data were downloaded from the TCPA database (https://www.tcpaportal.org/tcpa/). Tumor mutation data were downloaded from the TCGA database (https://portal.gdc.cancer.gov/), and we calculated the TMB through the “maftools” package of R language (version 4.1.1). Neoantigen data were obtained from TCIA database (https://www.tcia.at/). The TIDE algorithm was used to evaluate two mechanisms of tumor immune escape (T cell dysfunction and T cell exclusion)^28^.

### Statistical analysis

Spearman analysis was used to analyze the correlation between variables that did not obey the normal distribution. Wilcox test was used to compare two variables that did not follow the normal distribution, and *t* student test was used to compare two variables that meet the normal distribution. Cox regression was used to identify the independent prognosis factors.

## Results

### Identification of NK cells marker genes

**Figure 1:**
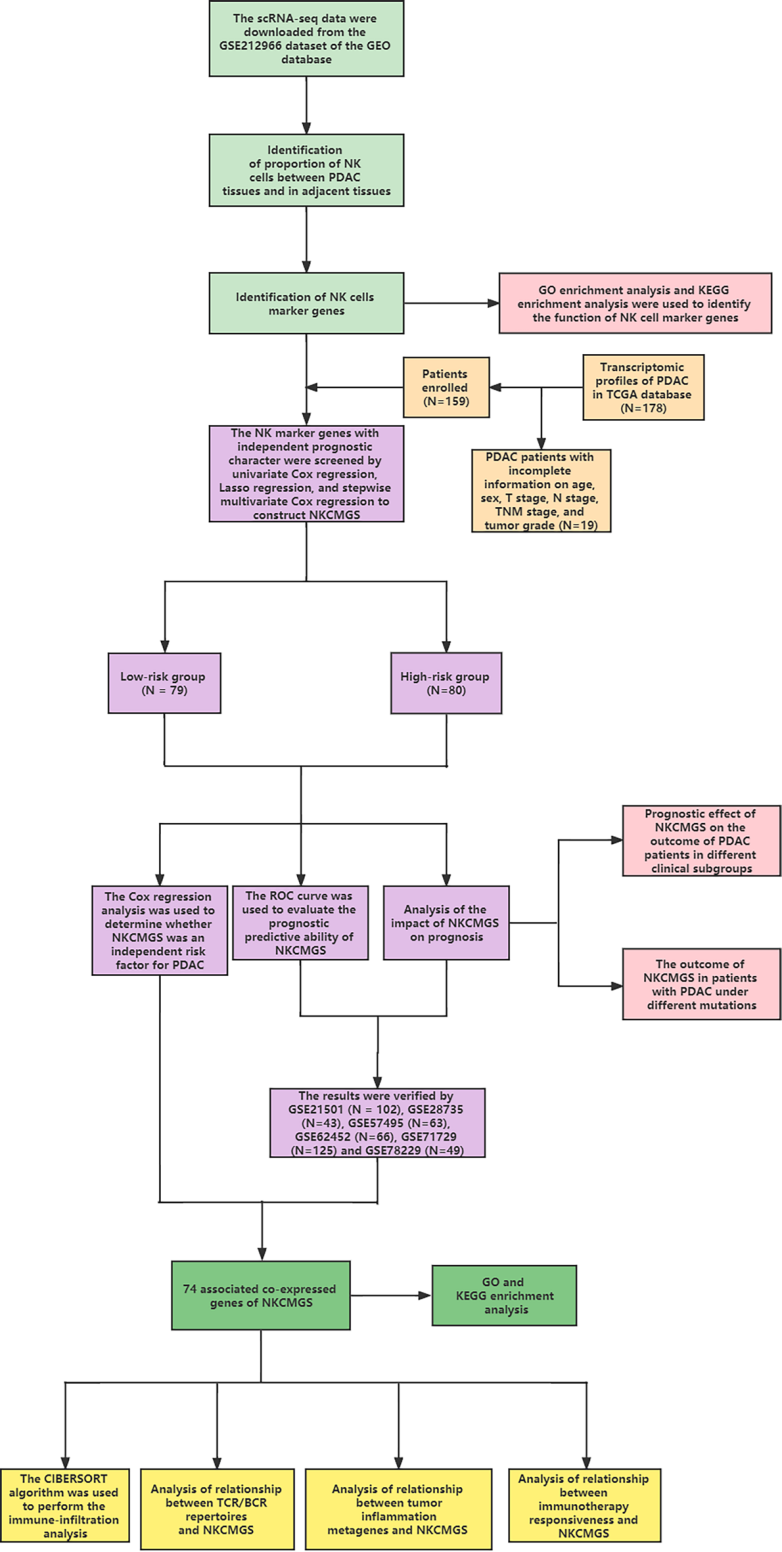
Flowchart of the study.

**Figure 2:**
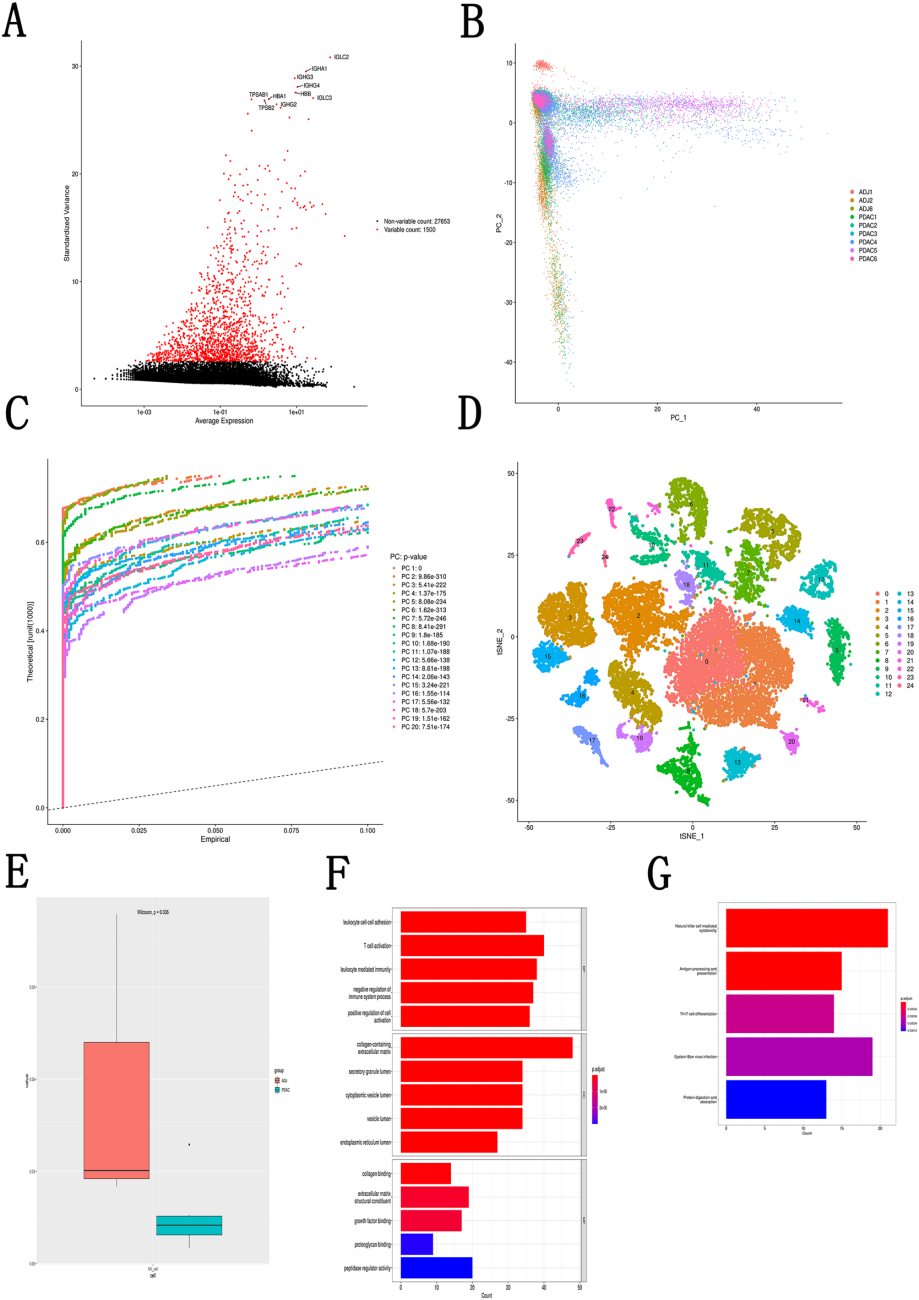
Identification of NK cells marker genes. (**A**) Scatter plot about the fluctuation situation of each gene; (**B**) PCA plot colored by different samples; (**C**) 20 PCs were identified based on P < 0.05; (**D**) t-SNE plot colored by various cell types; (**E**) Comparison of the proportion of NK cells in PDAC adjacent tissues and PDAC tissues; (**F**) GO enrichment analysis for NK marker genes; (**G**) KEGG enrichment analysis for NK marker genes.

The flowchart showed in Fig. 1. We generated expression profiles for a total of 57,167 cells from three PDAC adjacent tissues and six PDAC tissues. We selected the top 1500 representative genes for subsequent analysis (Fig. 2A). Subsequently, we performed the PCA dimensionality reduction on 57,167 cells by these 1500 genes (Fig. 2B). The top 20 representative PC were used for subsequent analysis (Fig. 2C). By t-SNE cluster analysis, we divided these cells into 25 cell clusters (Fig. 2D). The “SingleR” package helped us to annotate on these cell clusters (Table 1). Differential analysis of NK cells showed that NK cells were significantly less represented in PDAC tissues than in adjacent tissues (P < 0.05, Fig. 2E) which suggested that NK cells may be related to the occurrence and development of PDAC. Based on the results of the differential analysis, we further studied NK cell marker genes and the prognosis and immunity of PDAC patients. By representative genes distinguishing them from other cluster by NK cell cluster, we confirmed 373 NK cell marker genes (P < 0.01 and | log2 (fold change) | > 1).

**Table 1:** Annotation of the different cell clusters.

Cell type	Cell cluster
B_cells	9, 14
Chondrocytes	2
CMP	23
Endothelial_cells	8
Epithelial_cells	6, 10, 11, 21, 22
Fibroblasts	19
Macrophage	5
Monocyte	7, 17
Neurons	24
Neutrophils	3, 15
NK_cell	20
Smooth_muscle_cells	16
T_cells	0, 1, 13
Tissue_stem_cells	4, 12, 8

To further explore the function of these NK cell marker genes, we performed GO and KEGG analyses. The GO analysis showed that NK cell marker genes were abundant in functions such as leukocyte cell–cell adhesion, T cell activation, leukocyte mediated immunity, negative regulation of immune system process, and positive regulation of cell activation et al. function (Fig. 2F). The KEGG analysis showed that NK cell marker genes were enriched in functions such as natural killer cell mediated cytotoxicity, antigen processing and presentation, Th17 cell differentiation et al. function (Fig. 2G). The above results of enrichment analysis indicate that NK cell marker genes are closely related to immune regulation.

### Construction of NK cell marker genes prognostic signature

To construct the NK cell genes prognostic signature, we downloaded the transcriptome data and clinical data of PDAC patients from the TCGA database.

By univariate Cox regression analysis, we obtained 98 OS-associated NK marker genes (P < 0.05, Table S1). Subsequently, we performed Lasso regression with λ value above the lowest standard to screen out representative OS-associated NK cell marker genes. We obtained a total of 22 representative OS-associated NK cell marker genes (HIPK2, PSME1,NINL, CST3, TLE5, HDDC2, MT2A, KDM6B, PLAAT4, LDHA, EMP1, SKIL, IER3, DGKZ, PTMS, SLFN5, PPIC, MT1E, ASCL2, S100A16, EVL, PPP1CB) by Lasso regression (Fig. S1A,B). Through stepwise multivariate Cox regression, we obtained 7 NK marker genes with independent prognostic character to construct NKCMGS (Fig. S1C). According to the multivariate Cox coefficient, we calculated the risk score using the following formula: Risk score = (− 0.42632*NINL expression) + (− 0.54366*KDM6B expression) + (0.48692*PLAAT4 expression) + (0.65042*SKIL expression) + (− 0.42800*PPIC expression) + (0.29706*MT1E expression) + (− 0.26907*ASCL2 expression).

### The prognostic value of the NKCMGS

**Figure 3:**
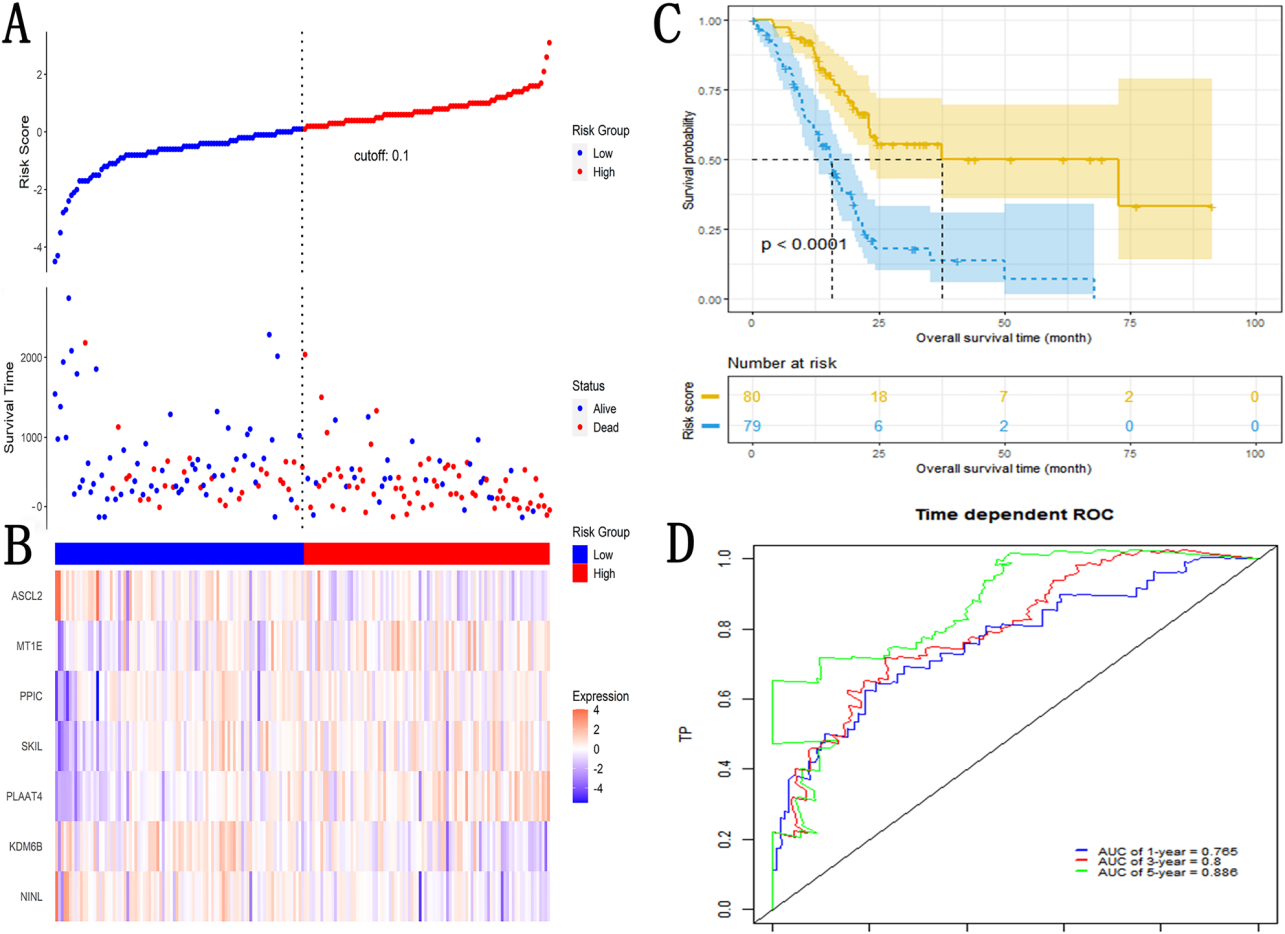
Construction of NK cell marker genes prognostic signature (**A**) Distribution of the vital status of PDAC patients in the different risk groups; (**B**) The heatmap represents the relationship between the expression of 7 NK marker genes constituting NKCMGS and vital status in PDAC patients; (**C**) Comparison of overall survival in different risk groups using Kaplan–Meier curves; (**D**) The ROC curve was used to evaluate the predictive ability of NKCMGS for the survival of 1-year, 3-year and 5-year in PDAC patients.

By median of risk score, we divided PDAC patients in TCGA into the high-risk group (N = 80) and the low-risk groups (N = 79). The risk plot indicates that a higher proportion of PDAC patients died in the high-risk group than the low-risk group (Fig. 3A). The heatmap represented the relationship of the 7 enrolled NK cell marker genes and risk score (Fig. 3B). The Kaplan–Meier analysis showed that the high-risk group had a worse prognosis than the low-risk group (P < 0.001, Fig. 3C). The results of ROCAUC (1-year: 0.765, 3-year: 0.800, 5-year: 0.885) showed that NKCMGS had a good predictive efficacy (Fig. 3D). Subsequently, we validated the prognostic value of NKCMGS for DFS by the same approach (Fig. S2). The results of Kaplan–Meier curve and ROCAUC implied that NKCGMS has good identification of high-risk PDAC patients.

**Figure 4:**
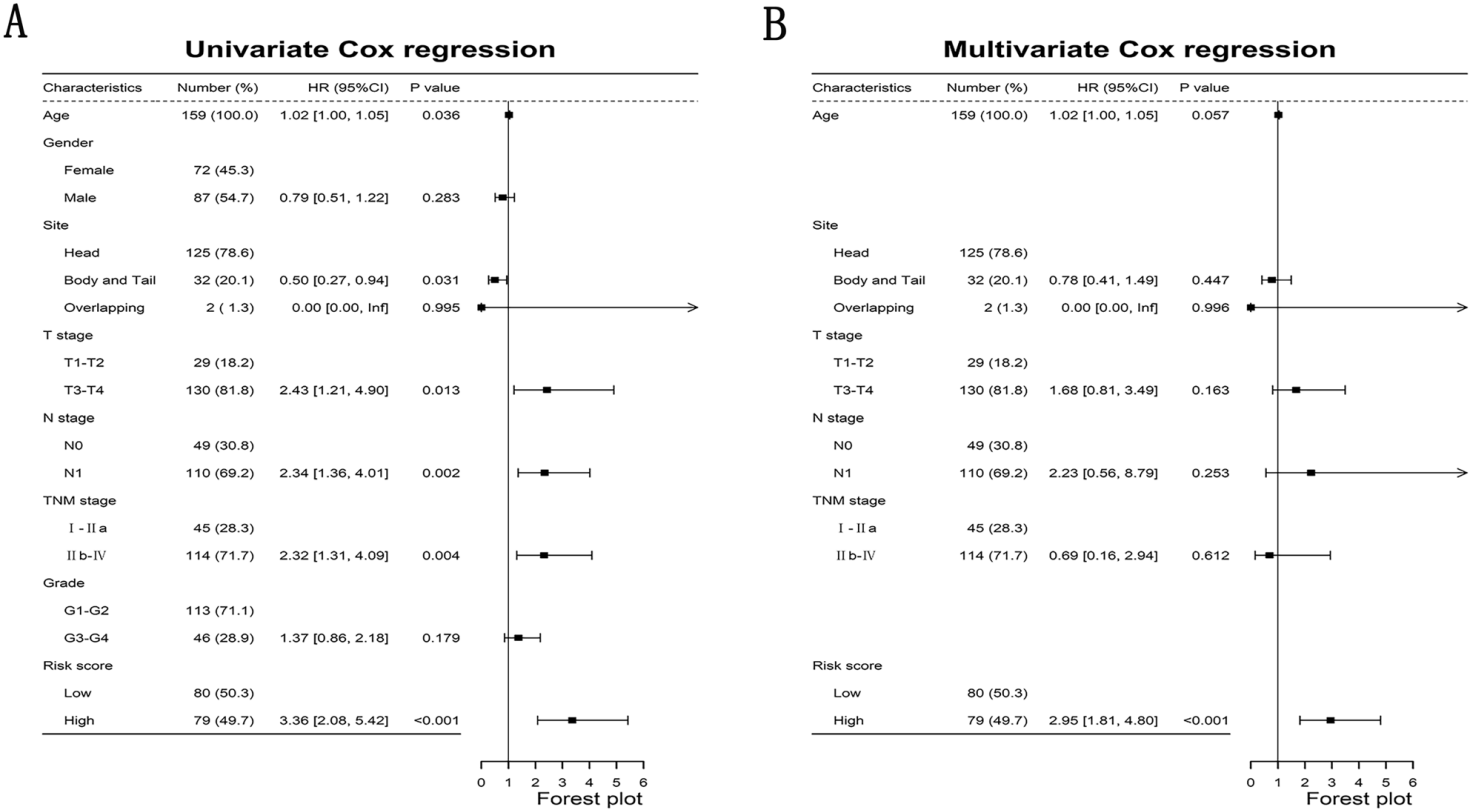
Identification of independent prognostic risk factors for PDAC. (**A**) Univariate Cox regression analysis was used to screen for risk factors for PDAC; (**B**) Multivariate Cox regression analysis was used to identify independent risk factors for PDAC.

After excluding PDAC patients with incomplete information about age, sex, T stage, N stage, TNM stage and grade (N = 19), Univariate and multivariate Cox regression were used to judge the independence of the NKCMGS. The results of univariate Cox regression showed that age (HR: 1.02 [1.00–1.05], P < 0.05), body and tail of pancreas (vs. head of pancreas, HR: 0.50 [0.27–0.94], P < 0.05), stage T3–T4 (vs. T1–T2, HR: 2.43 [1.21–4.90], P < 0.05), stage N1 (vs. N0, HR: 2.34 [1.36–4.01], P < 0.01), stage IIb–IV (vs. I–IIa, HR: 2.32 [1.31–4.09], P < 0.01), high risk score (vs. low, HR: 3.36 [2.08–5.42], P < 0.001) were prognostic factors of PDAC patients (Fig. 4A). The results of multivariate Cox regression showed that high risk score (vs. low, HR: 2.95 [1.81–4.80], P < 0.001) was the independent prognostic factor (Fig. 4B). This result represents that NKCMGS was the independent prognostic risk score for PDAC.

To verify the prognostic value of NKCMGS, we downloaded six GSE datasets (GSE21501, GSE28735, GSE57495, GSE62452, GSE71729 and GSE78229) from the GEO database. The baseline characteristics of these 6 GSE datasets are shown in Table S2. We calculated with the same method for the risk score values for each PDAC patient in the GSE dataset. Based on the optimal cut-off value of risk score, we divided PDAC patients into high-risk and low-risk groups. The Kaplan–Meier analysis showed that the prognosis in the high-risk group was significantly worse than that of the low-risk group (P < 0.01, Fig. S3A–F). The synthesis effects of the 6 GSE datasets were calculated by a Meta analysis (we selected a random effects model based on heterogeneity). The result of Meta analysis showed that NKCMGS (HR: 2.226 [1.903–2.55], P < 0.001) was associated with a worse prognosis in PDAC patients (Fig. S3G). We evaluated the prognostic prediction ability of NKCMGS with ROCAUC (Fig. S4): GSE21501 (1-year: 0.604, 3-year: 0.677, 5-year: 0.662), GSE28735 (1-year: 0.678, 3-year: 0.611, 5-year: NA), GSE57495 (1-year: 0.662, 3-year: 0.628, 5-year: 0.706), GSE62452 (1-year: 0.594, 3-year: 0.663, 5-year: 0.843), GSE71729 (1-year: 0.561, 3-year: 0.761, 5-year: 0.744), GSE78229 (1-year: 0.607, 3-year: 0.74, 5-year: 0.929), which suggested that NKCMGS is still able to stably identify high-risk patients in different datasets (GSE21501 for 1-year/3-year/5-year survival rate; GSE28735 for 1-year/3-year survival rate; GSE62452 for 3-year/5-year survival rate; GSE71729 for 3-year/5-year survival rate; GSE78229 for 1-year/3-year/5-year survival rate).

To explore the effect of NKCMGS in the different subgroups, we further analyzed the relationship between NKCMGS and patient outcomes in the different subgroups (age, gender, stage T, stage N, stage TNM, grade). The results showed that the OS in the high-risk group was shorter than that in the low-risk group (P < 0.05, Fig. S5). KRAS and TP53 are the most common mutations in PDAC, and previous studies have reported that KRAS and TP53 can promote the early metastasis of PDAC through synergistic effects and further negatively influence its prognosis^29^. In addition, somatic copy-number alternations (SCNA) is extremely common in cancer and drive cancer development^30^. Based on the importance of KRAS, TP53 and SCNA in pancreatic cancer, we performed subgroup analysis with the mutations of KRAS, TP 53 and the median SCNA load. The results of Kaplan–Meier analysis showed that the prognosis of the high-risk group was worse than that of the low-risk group except for the KRAS-WT subgroup (P < 0.05, Fig. S6). From the Kaplan–Meier curve of the KRAS-WT subgroup, the slope of the Kaplan–Meier curve changed at about 12 months, indicating a time bias. Therefore, we performed landmark analysis (12 month as landmark) to eliminate time bias and observed patient short-term (< 12 month) and long-term (> 12 month) outcomes. The results showed that in the KRAS-WT subgroup, the long-term prognosis of the high-risk group was significantly worse than that of the low-risk group (P < 0.001), but there was no significant difference in the short-term prognosis between the two groups (P > 0.05, Fig. S6D), which suggested that NKCMGS is still able to stably identify high-risk patients in different subgroup (NKCMGS was able to differentiate high-risk patients in the KRAS-MUT subgroup, TP53-WT subgroup, TP53-MUT subgroup, SCNA-low burden subgroup, SCNA-high burden subgroup, and was able to distinguish high-risk patients with long survival in the KRAS-WT subgroup).

### Biological pathways related to the NKCMGS

**Figure 5:**
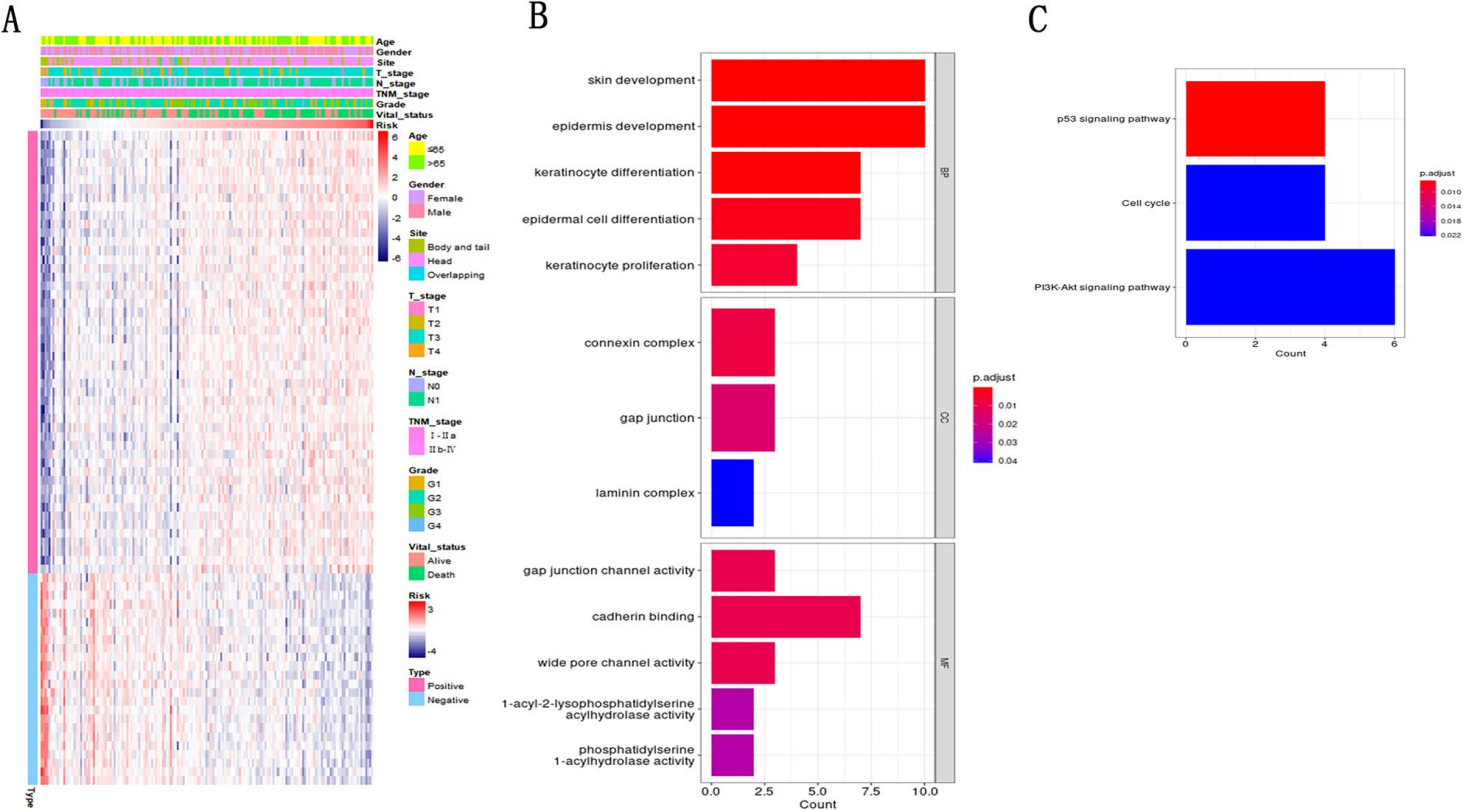
Functional exploration of correlated co-expressed genes of NKCMGS. (**A**) The heatmap shows the relationship between the 74 correlated co-expressed genes and the NKCMGS (|R| > 0.6, P < 0.001); (**B**) GO enrichment analysis for the 74 correlated co-expressed genes; (**C**) KEGG enrichment analysis for the 74 correlated co-expressed genes.

On the basis of the excellent prognostic value of NKCMGS, we further explored its potential mechanisms by TCGA database. Spearman correlation analysis screened 74 associated co-expressed genes (|R| > 0.6, P < 0.001), of which 24 genes were negatively correlated with NKCMGS and 50 genes were positively correlated with NKCMGS (Fig. 5A). GO enrichment analysis showed that the related co-expressed genes of NKCMGS were associated with skin development, epidermis development, keratinocyte differentiation, epidermal cell differentiation, and keratinocyte proliferation (Fig. 5B). KEGG enrichment analysis showed that the related co-expressed genes of NKCMGS were associated with P53 signaling pathway, cell cycle, PI3K-Akt signaling pathway (Fig. 5C), which implies that NKCMGS may influence tumor development and progression through multiple pathways.

### The relationship of NKCMGS with PDAC immune cell infiltration and the B/T cell receptor repertoire

**Figure 6:**
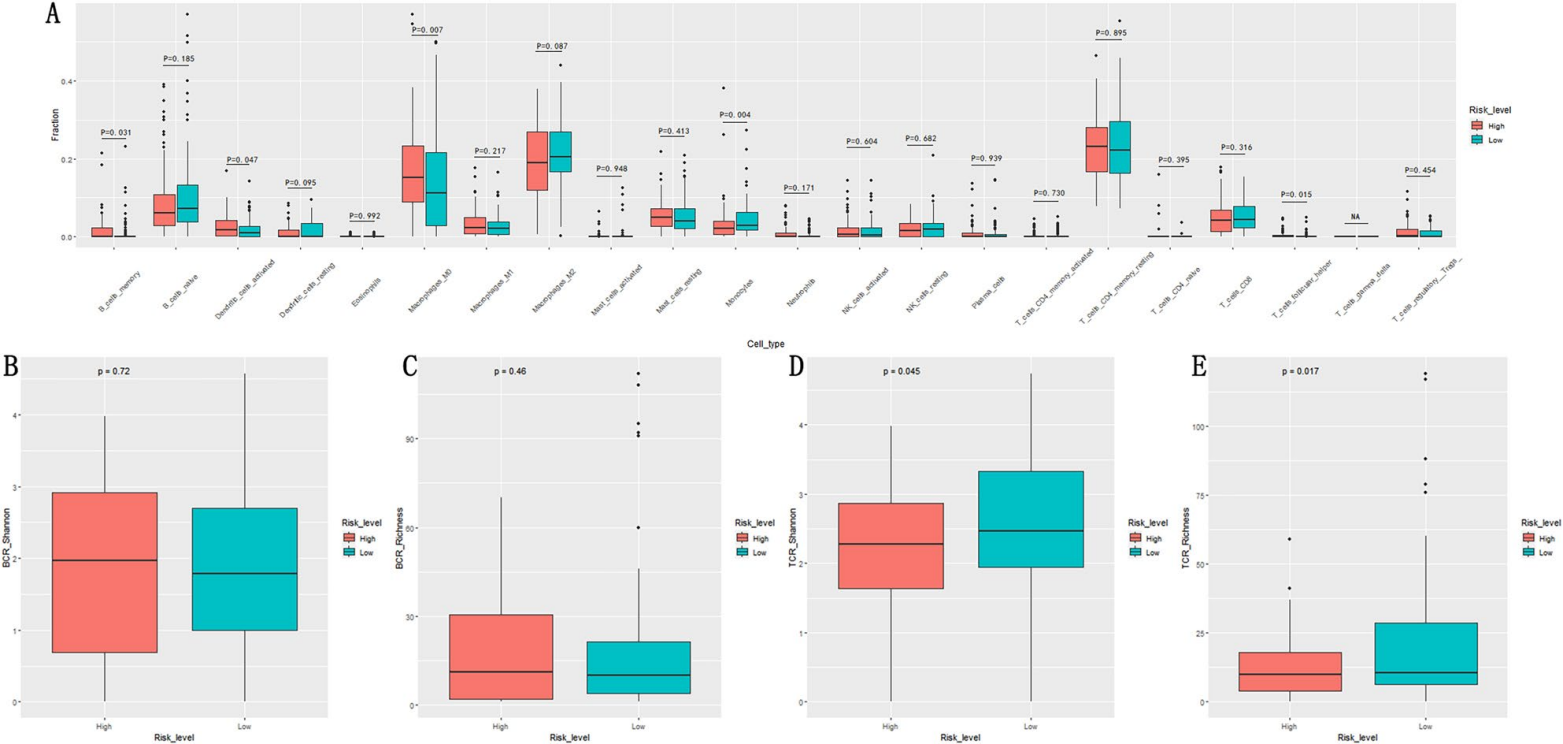
The relationship of NKCMGS and immune cell infiltration, diversity and richness of B/TCR repertoire. (**A**) The infiltration of 22 immune cells in different risk groups; (**B**) BCR Shannon index in different risk groups; (**C**) BCR Rishness index in different risk groups; (**D**) TCR Shannon index in different risk groups; (**E**) TCR Rishness index in different risk groups.

Given the importance of NK cells in tumor immunity, we analyzed the relationship between NKCMGS and PDAC immune cell infiltration by the CIBERSORT algorithm based on TCGA database. The results showed that the high-risk group had more B cells memory, dendritic cells activation, macrophage M0, and T cells follicular helper infiltration, but the monocytes had significantly less infiltration than the low-risk group (P < 0.05, Fig. 6A). Previous reports showed that the diversity and richness of the B/TCR repertoire is closely related to the adaptive immune function of B/T cells^31^. Therefore, we used the Shannon index and the Richness index to evaluate the diversity and richness of B/TCR repertoire, and tried to evaluate the adaptive immune function of B/T cells. We found that the diversity and richness of TCR repertoire were significantly lower in the high-risk group than in the low-risk group (P < 0.05, Fig. 6D,E), however, the diversity and richness of BCR repertoire were not significantly different between the two groups (P > 0.05, Fig. 6B,C), which may imply the overall low T cell function of the high-risk group patients despite their abundant T cells follicular helper infiltration.

### NKCMGS-related inflammatory features

**Figure 7:**
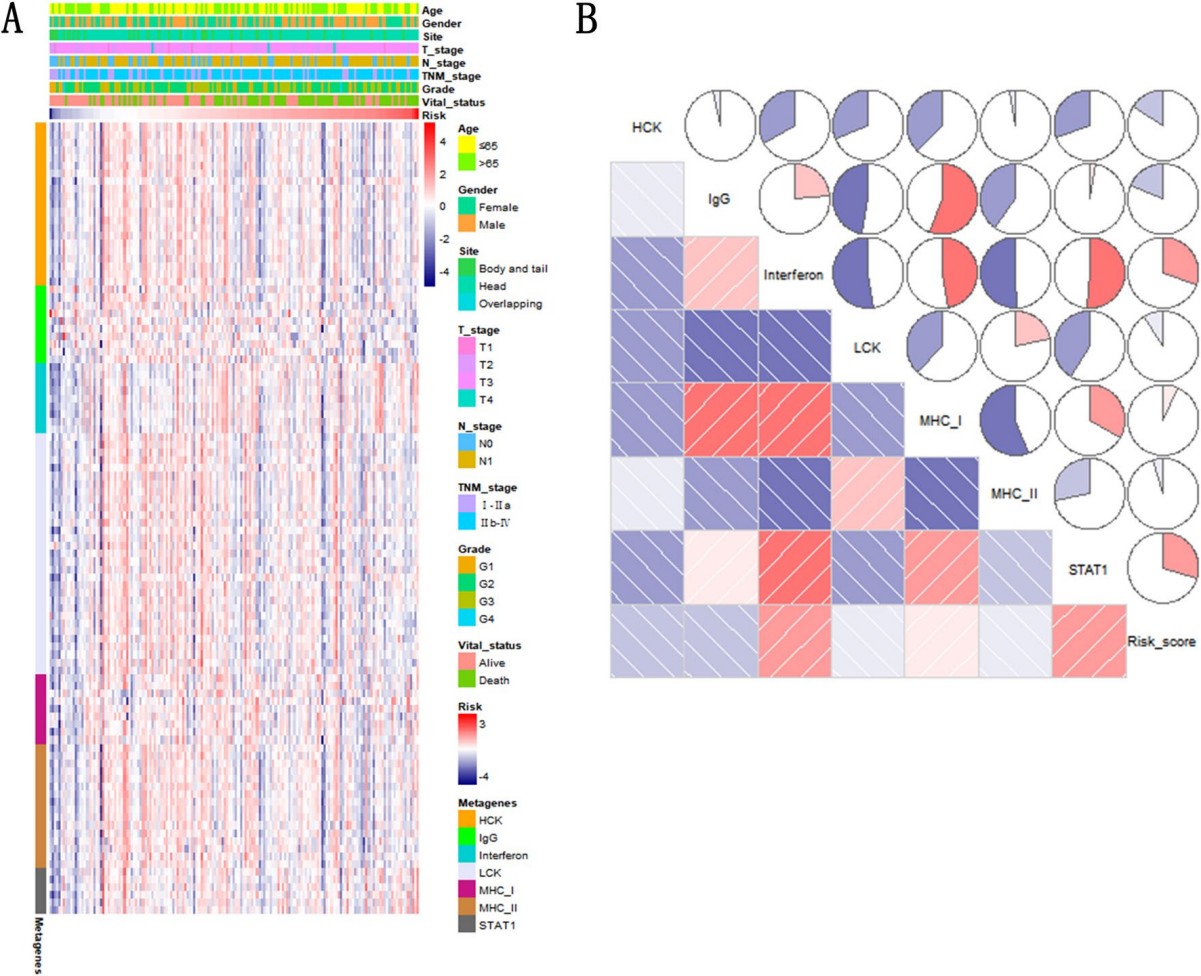
NKCMGS analysis of related inflammation characteristics. (**A**) Heatmap shows the relationship between risk score and inflammatory metagenes. (**B**) Correlogram shows the relationship between risk score and inflammatory metagene clusters.

We analyzed the relationship between NKCMGS and 7 metagenes (HCK, IgG, Interferon, LCK, MHC I, MHC II, STAT 1) representing different inflammations by TCGA database. The relationship between these metagenes and NKCMGS is shown in Fig. 7A. By using the GSVA algorithm, we obtained the expression level of 7 metagenes cluster. The Spearman correlation analysis was used to calculate the relationship between metagenes cluster and NKCMGS (Fig. 7B). The results showed that risk score was negatively associated with HCK, IgG, LCK, and MHC II and positively associated with Interferon, MHC I, and STAT1. It indicated that the process of presenting antigens from presenting-antigen cell to CD4+ T cell was impaired.

### NKCMGS and immunotherapy responsiveness

**Figure 8:**
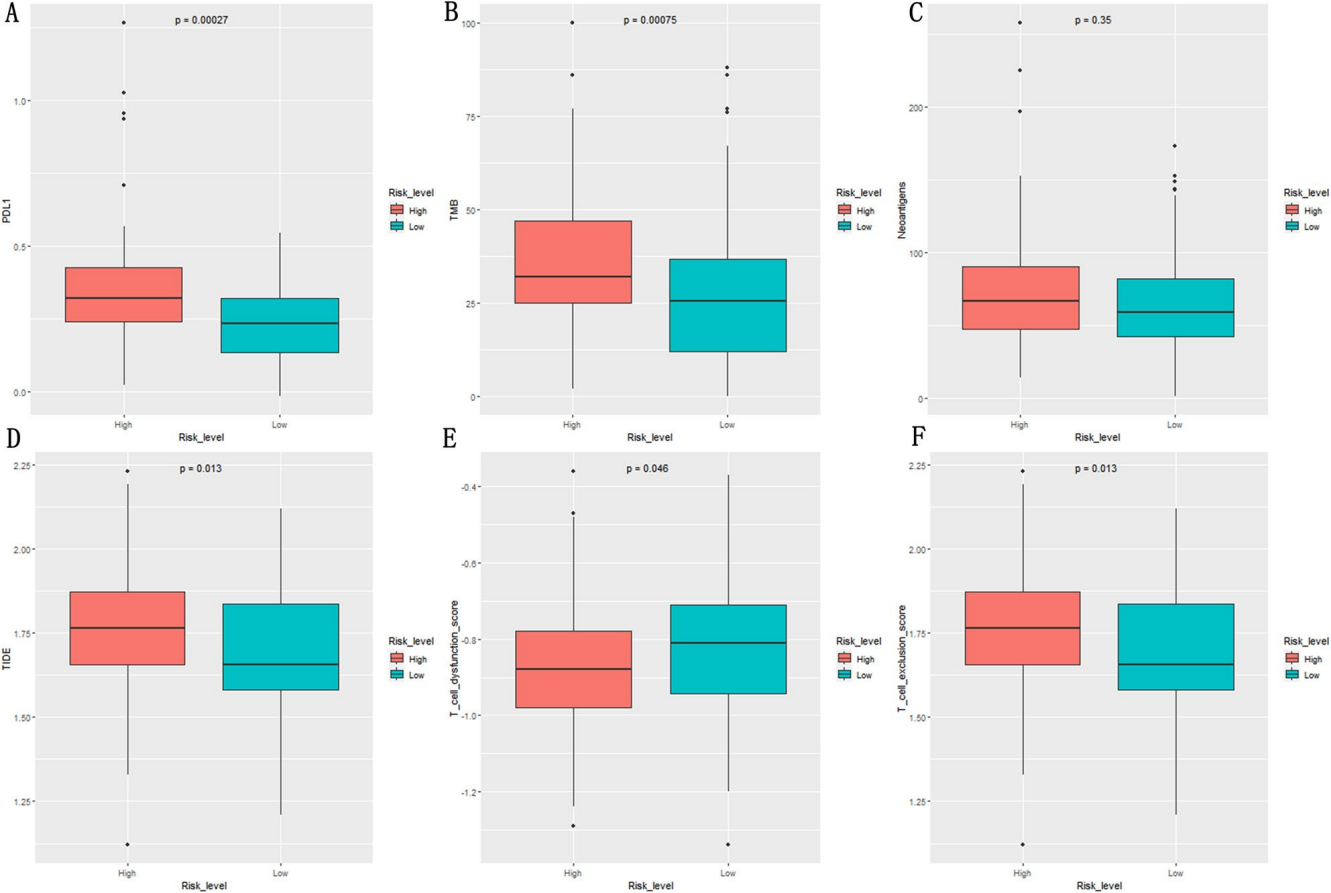
Comparison of immunotherapy-responsive biomarkers in different risk group. (**A**) PD-L1 protein expression in different risk groups; (**B**) TMB in different risk groups; (**C**) Number of neoantigens in different risk groups; (**D**) TIDE score in different groups; (**E**) T cell dysfunction score in different risk groups; (**F**) T cell exclusion score in different risk groups.

PD-L1 protein expression, TMB, neoantigens, and TIDE index are common predictors of immunotherapy response^28,32–34^. Therefore, we analyzed the relationship between NKCMGS and immunotherapy responsiveness in PDAC patients by comparing PD-L1 protein expression, TMB, neoantigens, and TIDE score in the high-risk and low-risk groups. The results showed that PD-L1 protein expression and TMB of the high-risk group had higher than low-risk group (P < 0.001, Fig. 8A,B). However, there was no significant difference in the level of neoantigens between the high-risk group and low-risk group (P > 0.05, Fig. 8C). The TIDE index indicates a poor responsiveness to immunotherapy in the high-risk group (P < 0.05, Fig. 8D). High-risk group had higher exclusion score and lower dysfunction score (P < 0.05, Fig. 8E,F). The high PD-L1 protein expression and TMB often suggest a better immunotherapy responsiveness and worse prognosis in high-risk group. Interestingly, the TIDE score, serve as a more accurate predictor than PD-L1 and TMB, showed a worse immunotherapy responsiveness in the high-risk group^35^.

## Discussion

TME acts as an extremely important contributor to the immunity and prognosis of PDAC^36^. NK cells, an important cellular components of the TME, were found to be significantly downregulated in PDAC in previous studies and affected the prognosis of PDAC patients^13–15^. Similarly, in our study, we also found a significant decrease in the proportion of NK cells in PDAC tissues compared to pancreatic cancer-adjacent tissues. In this study, we screened the marker genes of NK cells by analysis of scRNA-seq data. These marker genes were mainly enriched in immune-related functions, indicating the important position of NK cell marker genes in PDAC immunity. Based on the NK cell maker genes, we subsequently established the NK cell prognostic signature (NKCMGS) of PDAC patients using the TCGA database. We verified the prognostic value of NKCMGS in PDAC patients by the analysis of six independent GSE datasets. The results showed that NKCMGS is an independent risk factor for PDAC prognosis with good prognostic predictive value. GO analysis showed that NKCMGS-related genes were related to functions such as growth, differentiation and keratinization of epidermis. These genes associated with growth and differentiation and keratinization of epidermis usually also tend to promote the occurrence and development of malignant tumors^37^. Dong et al. considered genes upregulated by hypoxia in pancreatic cancer are enriched in keratinization, suggesting enhanced tight junction function, which may lead to chemotherapy resistance^38^. Ren et al. malignant tumor cells of found that pancreatic carcinoma exhibit keratinization properties^39^. KEGG analysis showed that NKCMGS-related genes are correlated with P53 signaling pathway, cell cycle, and PI3K-Akt signaling pathway. Previous studies have long confirmed the close relationship between P53 signaling pathway and cancer^40^. P53 is one of the most common mutations in PDAC and plays an influential part in the progression of PDAC^29^. Moreover, the dysregulation of the cell cycle is also one of the important causes of tumor cell progression. Study of Faleiro et al. demonstrated that the methylation of specific genes of PI3K-Akt signaling pathway is responsible for the recurrence and prognosis of pancreatic cancer^41^. All of these indicate a significant relationship between NKCMGS and the occurrence and development of PDAC. Consequently, we further validated the relationship between NKCMGS and the prognosis of PDAC patients in different clinical subgroups. The results indicated that NKCMGS had an impact the prognosis of PDAC patients in each clinical subgroups, which indicates a good prognostic value of NKCMGS. NKCMGS has higher stability and ability to identify high-risk patients compared to the model constructed by Zhang et al. based on neuroendocrine, metabolism-related genes and the iron death-related signature constructed by Chen et al.^42,43^.

In our study, NKCMGS is composed of a total of 7 NK cell marker genes (NINL, KDM6B, PLAAT4, SKIL, PPIC, MT1E, ASCL2). Previous studies have found an association between these genes and NK cell. Cribbs et al. found that combined knockdown of KDM6A and KDM6B histone demethylases reduces IFN-γ and TNF-α production in human NK cell populations^44^. Dassler-Plenker et al. also demonstrated functional PLAAT4 expression in naïve human NK cells and their direct activation upon PLAAT4 ligand (3pRNA) transfection^45^. Deubiquitinating enzymes removed K48-linked polyubiquitin chains on SKIL, SKI or Smad7, TGFβ signalling was dampened and TGFβ blocks NK cell-mediated adaptive immune system activation by downregulating the transcription factor T-bet, leading to reduced IFNγ expression^46^. Ppic could be involved in iNKT cell (a particular subset of αβ T lymphocytes that display properties of classical CD4 and CD8 αβ T lymphocytes and NK cells) differentiation and/or maintenance at the periphery^47^. The risk model of Jia et al. was established by the three m6A associated genes (including MT1E), which predicted lower resting NK cells levels in high-risk patients^48^. Li et al. found that ASCL 2 expression in colon cancer tissues showed a positive correlation with NK cell infiltration^49^. There are few studies on the relationship between NINL and NK cells, while the NINL knockout cells exhibit an impaired response to type I interferon^50^. Besides, Interferon α (the major type-I interferon) is able to enhance cytokine secretion, multifunctionality, degranulation, and cytotoxic potential of NK cell^51^. Among these NK cell marker genes, PLAAT4, SKIL, MT1E increase the prognostic risk of PDAC, while NINL, KDM6B, PPIC, and ASCL2 decrease the prognostic risk of PDAC. The Ninein-like protein encoded by NINL is a key factor for centrosome maturation, spindle formation, and chromosome segregation^52,53^. In previous studies, NINL was generally recognized as an oncogene, overexpressed in approximately 80% of breast and lung cancers^53,54^. However, Xiao et al. found that NINL-deficient mice were more susceptible to hepatomegaly and liver cancer, suggesting that this may be related to the promoting function of NINL on autophagy^55^. KDM6B, which can perform the dual effects of cancer promotion or suppressor base on different cell environment, is a histone demethylase specific for H3K27me3^56^. Yamamoto et al. found that KDM6B is highly expressed in pancreatic precancerous lesions, but its expression gradually decreased with increasing grade of pancreatic cancer^57^. Similarly, the study of Yamamoto et al. showed that nude mice after intrasplenic injection of pancreatic cancer cell lines (BxPC3) with KDM6B knocked down had a worse prognosis than nude mice after intrasplenic injection of BxPC3 cell lines without KDM6B knocked down^57^. PLAAT4 is a tumor suppressor induced by retinoids, and its high expression in cancer favors inhibiting the growth of cancer cells and promotes apoptosis^58^. However, PLAAT4 was highly expressed in PDAC tissues and related with worse prognosis of PDAC patients in the study of Li et al.^59^. SKIL is a key negative regulator of transforming growth factor β (TGF-β) signaling and is also an activator of p53^60^. Current studies showed that SKIL has both pro-cancer and tumor suppressor functions^61^. Liu et al. found that proliferation decreased and enhanced apoptosis in pancreatic cancer cell lines (sw1990) after silencing of SKIL^62^. PPIC encodes the cyclophilin C (Cyp-C), which is part of cyclophilin family^63^. PPIC has been used as a marker for circulating tumor cells in epithelial ovarian cancer^64^. The reports of PPIC in PDAC are quite limited. Metallothioneins (MT) are low-weight cysteine-rich proteins that are responsible for metal ion homeostasis in cells and therefore able to regulate cell proliferation and differentiation. MT can be classified into four subtypes: MT1E, MT2A, MT3, and MT4^65^. The study of Demidenko et al. showed that MT1E is expressed at low levels in prostate cancer, and low expression of MT1E is associated with poor prognosis in prostate cancer^66^. However, MT1E expression in glioma was able to promote the migration and invasion effects of glioma^67,68^. Xie et al. found that overexpression of MT1E was associated with drug resistance in pancreatic cancer^69^. The basic helix-loop helical transcription factor (TF) gene ASCL2 is a transcriptional target of Wnt signaling^70^. ASCL2 was highly expressed in breast, colon, gastric, lung, head and neck, ovarian, and testicular cancers, while is less expressed in sarcoma, melanoma, brain, and prostate cancers^71^. However, there are few studies on ASCL2 in PDAC. Previous studies suggested that these NK cell marker genes involve immune regulatory processes (For example, NINL and KDM6B in gastric cancer^72,73^, PLAAT4 in pancreatic carcinoma^59^, SKIL in non-small cell lung cancer^74^, MT1E in liver cancer^75^, ASCL2 in colorectal cancer)^76^ in solid malignant tumors. The identification of NKCMGS of PDAC provides a potential target for therapeutic and mechanistic studies of PDAC.

Considering the close relationship between tumor immune infiltration and prognosis^77^, we further investigated the relationship of NKCMGS with the immune features of PDAC. According to the results of CIBERSORT analysis, we found more B cells memory, dendritic cells activated, macrophage M0, and T cells follicular helper infiltration in the high-risk group, but significantly less monocyte infiltration than in the low-risk group. The memory B cells in PDAC are usually associated with the tertiary lymphatic structure (TLS) of PDAC, which usually suggests longer OS and PFS^78,79^. Dendritic cells are considered to be the most potent antigen-presenting cells and plays an important role in adaptive immunity^80^. Enrichment of follicular helper T (Tfh) cells implies the formation of a TLS^81^. Good immune cell infiltration often means that the high-risk group should have a good prognosis. Interestingly, high-risk group did not show a favorable prognosis. The diversity and richness of the B/TCR gene repertoire closely follows the adaptive immune function of B/T cells^31^. We found that high-risk group had lower TCR diversity and richness than low-risk group. This suggests that the overall T cells of high-risk group function was weaker than low-risk group. To some extent, this could explain the fact that high-risk group has a favorable immune infiltrate but a worse prognosis. Similarly, the analysis of inflammatory metagenes showed a negative relationship between risk score and monocyte/macrophage function, B cell function, T cell function, and process of antigen presentation to T cells (HCK, IgG, LCK, MHC-II). The above suggests that TME of PDAC may be one of the mechanisms by which NKCMGS influences the prognosis.

PD-L1 protein expression, TMB and neoantigens are common predictors of immunotherapy response^32–34^. TIDE is a more accurate predictor than PD-L1 protein expression, TMB and neoantigens^35^. We found that high-risk group had a higher PD-L1 protein expression and TMB, which often suggests that high-risk group should exhibit excellent immunotherapy responsiveness and worse prognosis. Interestingly, the high TIDE score showed poorer immunotherapy responsiveness in the high-risk group. According to T cell exclusion score, we found that phenomenon of “immune exclusion” in the high-risk group. The report of Pai et al. showed that chemoattraction recruits T cells to the tumor microenvironment and antigen stimulation promotes their persistence, which is the basis of good immunotherapy responsiveness^82^. In our study, in the high-risk group, despite the presence of favorable antigenic stimulation (the PD-L1 protein expression and TMB of high-risk group is higher than low-risk group), due to the presence of mechanical and/or functional barrier, T cells may have difficulty entering the tumor parenchyma, and it only remained in the tumor stroma (the exclusion score of high-risk group is higher than low-risk group). This phenomenon can also be seen in other malignancies. Zhong et al. divided renal clear cell carcinomas into 2 subgroups (C1 and C2), and the TIDE score, TMB, and PD-L1 expression were all higher in the C1 subgroup than in the C2 subgroup^83^. In the study of Peng et al., TMB in colorectal cancer high-risk group was slightly higher than that in the low-risk group and TIDE score of high-risk group was higher than low-risk group^84^. The study of Pan et al. showed that lung adenocarcinoma low-risk group has both high PD-L1 expression and TIDE scores^35^. This suggests that the PDAC patients with high risk score of NKCMGS have the worse immunotherapy responsiveness. Therefore, clinicians can individualize interventions based on the patient's risk score. Since patients with low-risk score have a higher therapeutic response to immunotherapy, clinicians can select patients with low-risk scores to receive more active immunotherapy regimen. Besides, patients with high-risk scores tend to die earlier, and clinicians should pay more attention to these high-risk patients.

This study has some imperfections: (1) This study is a retrospective study based on the database, which is likely to cause bias. The predictive power of NKCMGS still needs to be verified in large-scale prospective studies; (2) This study about the relationship of NKCMGS and the immune function of PDAC patients is only based on the indirect assessment of predictive indicators; (3) Further validation in in vitro and in vivo experiments is still needed.

## Conclusions

In summary, we combined scRNA-seq and bulk RNA-seq analysis to establish a NKCMGS consisting of 7 NK cell marker genes. NKCMGS has a good prognosis and the predictive ability of the immunotherapy response. Through detected the expression of 7 NK cell marker genes in PDAC tissue, clinicians can use NKCMGS to build models for individualized prediction of pancreatic cancer patients, and it can also serve as a potential target for targeted therapy of pancreatic cancer. This study concludes that the predictive value of NKCMGS for prognosis and immunotherapy responsiveness of PDAC patients, also provides a reference for the individualization of immunotherapy in PDAC patients.

### Supplementary Information

Supplementary Information.

## Data Availability

The data used to support the findings of this study are available from GEO database (https://www.ncbi.nlm.nih.gov/geo/, number: GSE212966, GSE21501, GSE28735, GSE57495, GSE62452, GSE71729 and GSE78229), UCSC Xena database (http://xena.ucsc.edu/, number: TCGA-PAAD), TCPA database (https://www.tcpaportal.org/tcpa/, number: Pancreatic adenocarcinoma (PAAD)), and TCGA database (https://portal.gdc.cancer.gov/, number: PAAD), TCIA database (https://www.tcia.at/, number: PAAD). These are all open-access databases.
