# Supplementary material for: Combining single-cell and bulk RNA sequencing, NK cell marker genes reveal a prognostic and immune status in pancreatic ductal adenocarcinoma
Source: Sci Rep. 2024 Jul 1;14:15037. doi: 10.1038/s41598-024-65917-1 (PMC11217423; doi:10.1038/s41598-024-65917-1)
Supplement: Supplementary file 1 — Supplementary Information. [file 41598_2024_65917_MOESM1_ESM.docx]

**Table S1 Univariate Cox regression analysis was used to screen for the OS-related NK marker genes**

| **ID** | **Genes** | **HR** | **P** |
| --- | --- | --- | --- |
| 1 | CFH | 1.1280[0.9704-1.311] | 0.117 |
| 2 | CD99 | 0.8285[0.6333-1.084] | 0.17 |
| 3 | ITGAL | 1.007[0.8945-1.133] | 0.914 |
| 4 | TNFRSF12A | 1.31[1.089-1.574] | 0.00407 |
| 5 | MATK | 0.8633[0.7282-1.023] | 0.0905 |
| 6 | IL32 | 1.362[1.13-1.643] | 0.00121 |
| 7 | TRAF3IP3 | 0.9965[0.8783-1.131] | 0.957 |
| 8 | CD9 | 1.235[1.049-1.453] | 0.0113 |
| 9 | ABHD5 | 1.262[0.9793-1.625] | 0.0721 |
| 10 | PLAUR | 1.277[1.077-1.513] | 0.00485 |
| 11 | DCN | 1.093[0.9564-1.249] | 0.192 |
| 12 | TYROBP | 1.048[0.8897-1.234] | 0.576 |
| 13 | RUNX3 | 1.039[0.9027-1.195] | 0.596 |
| 14 | BIRC3 | 1.258[1.096-1.444] | 0.00109 |
| 15 | VIM | 1.129[0.9168-1.391] | 0.253 |
| 16 | CD44 | 1.485[1.17-1.884] | 0.00113 |
| 17 | GRN | 0.8618[0.6392-1.162] | 0.329 |
| 18 | TIMP2 | 1.123[0.9102-1.385] | 0.28 |
| 19 | MYOM2 | 0.8898[0.7475-1.059] | 0.189 |
| 20 | VCAN | 1.162[1.024-1.319] | 0.0196 |
| 21 | CAPG | 1.321[1.104-1.581] | 0.00239 |
| 22 | CYBA | 1.181[0.9968-1.4] | 0.0545 |
| 23 | PRDM1 | 1.231[1.012-1.498] | 0.0378 |
| 24 | MXD1 | 1.251[1.044-1.499] | 0.0151 |
| 25 | YBX3 | 1.418[1.137-1.769] | 0.00194 |
| 26 | VMP1 | 1.096[0.8881-1.352] | 0.394 |
| 27 | CASP8 | 1.387[1.103-1.744] | 0.00508 |
| 28 | HIPK2 | 0.8274[0.686-0.9979] | 0.0475 |
| 29 | RASSF1 | 1.029[0.7167-1.478] | 0.876 |
| 30 | ACTN1 | 1.227[0.9473-1.589] | 0.121 |
| 31 | TBX21 | 1.016[0.8866-1.165] | 0.818 |
| 32 | TXK | 0.9859[0.8325-1.167] | 0.869 |
| 33 | TTC38 | 1.099[0.8283-1.457] | 0.514 |
| 34 | FBLN1 | 1.091[0.9653-1.233] | 0.163 |
| 35 | CST7 | 1.056[0.9128-1.223] | 0.462 |
| 36 | PPP2R5C | 0.9994[0.704-1.419] | 0.997 |
| 37 | HSP90AA1 | 1.134[0.87-1.479] | 0.352 |
| 38 | APLP2 | 1.344[1.034-1.747] | 0.0269 |
| 39 | CD59 | 1.331[0.9888-1.791] | 0.0594 |
| 40 | DNAJA1 | 0.9032[0.6761-1.207] | 0.491 |
| 41 | PPP1R15A | 1.211[0.9643-1.521] | 0.0995 |
| 42 | FTL | 1.021[0.787-1.324] | 0.878 |
| 43 | MMP2 | 1.183[1.041-1.344] | 0.00989 |
| 44 | PTPN4 | 1.035[0.7868-1.362] | 0.805 |
| 45 | DYNLL1 | 0.9136[0.6235-1.339] | 0.643 |
| 46 | RGS1 | 1.111[0.972-1.271] | 0.122 |
| 47 | LYZ | 1.051[0.9599-1.151] | 0.281 |
| 48 | CPA1 | 1.023[0.988-1.059] | 0.202 |
| 49 | CCDC80 | 1.101[0.9816-1.236] | 0.1 |
| 50 | PSME1 | 1.485[1.066-2.07] | 0.0196 |
| 51 | EZR | 1.393[1.129-1.719] | 0.00198 |
| 52 | CREM | 0.9047[0.6965-1.175] | 0.453 |
| 53 | HSP90AB1 | 1.089[0.7787-1.523] | 0.619 |
| 54 | GADD45B | 0.9478[0.7566-1.187] | 0.641 |
| 55 | YPEL1 | 0.9262[0.7322-1.171] | 0.522 |
| 56 | TIMP3 | 1.067[0.9361-1.216] | 0.332 |
| 57 | GZMH | 1.087[0.953-1.24] | 0.213 |
| 58 | GZMB | 1.16[1.014-1.328] | 0.0307 |
| 59 | HIF1A | 1.2[0.9617-1.497] | 0.107 |
| 60 | NFKBIA | 1.086[0.825-1.429] | 0.558 |
| 61 | NINL | 0.8063[0.6741-0.9645] | 0.0185 |
| 62 | CTSZ | 1.174[0.9289-1.485] | 0.179 |
| 63 | MYL9 | 1.07[0.9087-1.261] | 0.416 |
| 64 | CST3 | 0.6971[0.515-0.9436] | 0.0195 |
| 65 | APMAP | 0.7696[0.5566-1.064] | 0.113 |
| 66 | MYL12A | 1.424[1.133-1.79] | 0.00242 |
| 67 | TIMP1 | 1.011[0.8202-1.246] | 0.918 |
| 68 | RGCC | 0.8493[0.6806-1.06] | 0.148 |
| 69 | TSC22D1 | 1.03[0.7701-1.377] | 0.843 |
| 70 | CORO1A | 0.987[0.8521-1.143] | 0.862 |
| 71 | COTL1 | 1.251[0.9951-1.572] | 0.0551 |
| 72 | CSK | 1.001[0.7544-1.328] | 0.995 |
| 73 | FCGRT | 0.7766[0.568-1.062] | 0.113 |
| 74 | TLE5 | 0.5322[0.3584-0.7901] | 0.00176 |
| 75 | NKG7 | 1.032[0.8883-1.199] | 0.682 |
| 76 | NAMPT | 1.362[1.075-1.724] | 0.0104 |
| 77 | HSPB1 | 1.235[0.982-1.554] | 0.0712 |
| 78 | RARRES2 | 1.19[0.9903-1.429] | 0.0634 |
| 79 | AHR | 1.315[1.085-1.594] | 0.00528 |
| 80 | AEBP1 | 1.118[0.9654-1.294] | 0.137 |
| 81 | AKNA | 0.955[0.7907-1.153] | 0.632 |
| 82 | PTGDS | 1.012[0.9071-1.129] | 0.834 |
| 83 | ZFAND5 | 0.9626[0.713-1.3] | 0.804 |
| 84 | ACTA2 | 1.093[0.9373-1.274] | 0.257 |
| 85 | RGS9 | 0.7775[0.6725-0.8989] | 0.000677 |
| 86 | PFN1 | 1.15[0.8128-1.626] | 0.431 |
| 87 | LGALS3BP | 1.035[0.786-1.363] | 0.806 |
| 88 | ABI3 | 0.9008[0.7403-1.096] | 0.297 |
| 89 | COL1A1 | 1.138[1.009-1.284] | 0.0356 |
| 90 | SLC9A3R1 | 0.9997[0.8001-1.249] | 0.998 |
| 91 | PMP22 | 1.157[0.9634-1.389] | 0.119 |
| 92 | B3GAT1 | 0.8383[0.7529-0.9334] | 0.00129 |
| 93 | MDK | 1.131[0.9018-1.418] | 0.287 |
| 94 | SELPLG | 1.158[0.9907-1.353] | 0.0654 |
| 95 | BIN2 | 1.018[0.8795-1.177] | 0.815 |
| 96 | MGP | 0.9841[0.8589-1.128] | 0.817 |
| 97 | GNPTAB | 1.139[0.8748-1.483] | 0.333 |
| 98 | KLRB1 | 0.9853[0.8667-1.12] | 0.82 |
| 99 | HDDC2 | 0.6852[0.4701-0.9988] | 0.0493 |
| 100 | SOD2 | 1.217[1.002-1.479] | 0.0477 |
| 101 | CCND3 | 0.9462[0.7053-1.269] | 0.712 |
| 102 | VEGFA | 1.093[0.8986-1.329] | 0.374 |
| 103 | SPARC | 1.132[0.9671-1.325] | 0.123 |
| 104 | HES1 | 1.327[1.047-1.683] | 0.0195 |
| 105 | ZAP70 | 0.9417[0.8267-1.073] | 0.366 |
| 106 | SPTBN1 | 1.205[0.9439-1.54] | 0.134 |
| 107 | FN1 | 1.17[1.034-1.324] | 0.0131 |
| 108 | GNLY | 1.108[0.9441-1.299] | 0.21 |
| 109 | HSPE1 | 1.197[0.9113-1.573] | 0.196 |
| 110 | ID2 | 0.9312[0.7052-1.23] | 0.615 |
| 111 | PLEK | 1.034[0.9134-1.17] | 0.599 |
| 112 | AAK1 | 0.9235[0.7003-1.218] | 0.573 |
| 113 | RGS2 | 1.102[0.9308-1.304] | 0.26 |
| 114 | CD48 | 1.008[0.901-1.127] | 0.894 |
| 115 | RAB29 | 1.042[0.8033-1.351] | 0.758 |
| 116 | ID3 | 0.9956[0.7867-1.26] | 0.971 |
| 117 | PRDX1 | 1.379[1.05-1.812] | 0.0209 |
| 118 | CNN3 | 1.093[0.8833-1.353] | 0.413 |
| 119 | TNFAIP3 | 1.108[0.9331-1.316] | 0.241 |
| 120 | SGK1 | 1.145[0.9517-1.377] | 0.151 |
| 121 | CCN2 | 1.032[0.8988-1.185] | 0.655 |
| 122 | VAMP8 | 1.359[1.048-1.761] | 0.0205 |
| 123 | ELL2 | 0.839[0.7011-1.004] | 0.0554 |
| 124 | YPEL5 | 1.078[0.7583-1.532] | 0.676 |
| 125 | DUSP1 | 1.054[0.8905-1.247] | 0.542 |
| 126 | CYSTM1 | 1.043[0.8653-1.256] | 0.661 |
| 127 | PLXDC2 | 1.106[0.9549-1.281] | 0.179 |
| 128 | HSPH1 | 1.056[0.8287-1.347] | 0.658 |
| 129 | EGR1 | 1.003[0.8681-1.159] | 0.969 |
| 130 | CALD1 | 1.12[0.9576-1.311] | 0.156 |
| 131 | SRGN | 1.037[0.8936-1.203] | 0.633 |
| 132 | NR4A1 | 1.011[0.8885-1.151] | 0.864 |
| 133 | LRP1 | 1.119[0.9259-1.352] | 0.245 |
| 134 | PMEPA1 | 1.234[1.052-1.447] | 0.00985 |
| 135 | CDKN1A | 1.242[0.9436-1.636] | 0.122 |
| 136 | SOX4 | 1.383[1.109-1.724] | 0.00397 |
| 137 | MT2A | 1.212[1.019-1.441] | 0.0297 |
| 138 | PTGER2 | 1.115[0.9597-1.296] | 0.155 |
| 139 | C3 | 1.111[0.9923-1.244] | 0.0679 |
| 140 | FOSB | 0.98[0.8906-1.078] | 0.678 |
| 141 | HCST | 0.8954[0.749-1.07] | 0.225 |
| 142 | RAP1B | 1.334[1.001-1.777] | 0.0493 |
| 143 | KLF2 | 1.045[0.8915-1.225] | 0.586 |
| 144 | RAC2 | 1.247[1.042-1.492] | 0.0162 |
| 145 | PALLD | 1.126[0.9387-1.351] | 0.201 |
| 146 | ABHD17A | 0.5742[0.4355-0.757] | 8.38e-05 |
| 147 | SAT1 | 1.382[1.085-1.76] | 0.00881 |
| 148 | APOE | 0.9218[0.811-1.048] | 0.213 |
| 149 | JUND | 0.7429[0.5788-0.9536] | 0.0196 |
| 150 | LSP1 | 1.072[0.9276-1.238] | 0.348 |
| 151 | ZNF331 | 0.896[0.7642-1.05] | 0.176 |
| 152 | SYNE1 | 0.8882[0.75-1.052] | 0.169 |
| 153 | LGALS3 | 1.369[1.145-1.638] | 0.000581 |
| 154 | DNAJB1 | 1.195[0.9575-1.491] | 0.115 |
| 155 | SERPINF1 | 1.085[0.9476-1.242] | 0.238 |
| 156 | KDM6B | 0.7257[0.5567-0.9459] | 0.0177 |
| 157 | PLAAT4 | 1.436[1.212-1.701] | 2.91e-05 |
| 158 | GIMAP4 | 0.9986[0.8489-1.175] | 0.986 |
| 159 | LDHA | 1.66[1.295-2.127] | 6.14e-05 |
| 160 | IL6ST | 1.023[0.8292-1.262] | 0.832 |
| 161 | EMP1 | 1.246[1.063-1.461] | 0.00655 |
| 162 | KLRD1 | 1.1[0.9543-1.268] | 0.188 |
| 163 | KLRC1 | 1.066[0.9237-1.23] | 0.382 |
| 164 | ETS1 | 1.153[0.9465-1.404] | 0.158 |
| 165 | CTSL | 1.014[0.8222-1.25] | 0.898 |
| 166 | GLUL | 1.157[0.919-1.458] | 0.214 |
| 167 | CHST12 | 0.6358[0.4818-0.839] | 0.00137 |
| 168 | GPNMB | 1.079[0.9539-1.221] | 0.226 |
| 169 | MYO1G | 1.016[0.8713-1.185] | 0.837 |
| 170 | LIMD2 | 0.9254[0.787-1.088] | 0.348 |
| 171 | SKIL | 1.327[1.07-1.646] | 0.01 |
| 172 | TXN | 1.435[1.098-1.877] | 0.00829 |
| 173 | KLF4 | 1.182[0.9922-1.408] | 0.0612 |
| 174 | ARPC5L | 1.024[0.729-1.439] | 0.891 |
| 175 | IER3 | 1.249[1.059-1.473] | 0.00813 |
| 176 | CLPS | 1.019[0.9824-1.057] | 0.315 |
| 177 | FGFBP2 | 0.8797[0.7549-1.025] | 0.1 |
| 178 | SDCBP | 1.28[0.9825-1.669] | 0.0674 |
| 179 | THBS1 | 1.117[0.9881-1.263] | 0.0769 |
| 180 | PHLDA1 | 1.444[1.129-1.847] | 0.00344 |
| 181 | LUM | 1.137[0.9954-1.298] | 0.0584 |
| 182 | GPR65 | 1.029[0.8963-1.182] | 0.683 |
| 183 | TPM1 | 1.224[1.001-1.498] | 0.0494 |
| 184 | SKAP1 | 1.006[0.8843-1.145] | 0.924 |
| 185 | IGFBP4 | 1.04[0.8488-1.274] | 0.707 |
| 186 | IFITM3 | 1.306[1.047-1.63] | 0.0181 |
| 187 | COL6A1 | 1.122[0.9487-1.327] | 0.179 |
| 188 | COL6A2 | 1.075[0.9079-1.272] | 0.402 |
| 189 | APP | 1.074[0.8215-1.405] | 0.601 |
| 190 | MYO1F | 0.9883[0.8422-1.16] | 0.885 |
| 191 | EFHD2 | 1.191[0.9478-1.495] | 0.134 |
| 192 | SH3BGRL3 | 1.394[1.094-1.777] | 0.00723 |
| 193 | SYTL1 | 1.027[0.8347-1.265] | 0.799 |
| 194 | CELA3A | 1.021[0.986- 1.057] | 0.244 |
| 195 | HSPG2 | 1.08[0.9214-1.267] | 0.341 |
| 196 | CCN1 | 1.094[0.956-1.252] | 0.192 |
| 197 | CD53 | 1.054[0.9267-1.198] | 0.425 |
| 198 | MCL1 | 1.298[1.003-1.681] | 0.0475 |
| 199 | HSPD1 | 1.242[0.9377-1.646] | 0.131 |
| 200 | ARL6IP5 | 1.271[0.9477- 1.705] | 0.109 |
| 201 | NFKBIZ | 1.12[0.9452-1.328] | 0.19 |
| 202 | LYAR | 1.223[0.9197-1.627] | 0.166 |
| 203 | PLAC8 | 1.186[1.047-1.344] | 0.00718 |
| 204 | SFRP2 | 1.127[1.022-1.243] | 0.0165 |
| 205 | GZMA | 1.024[0.8863-1.182] | 0.752 |
| 206 | IL2RG | 1.102[0.9799-1.24] | 0.105 |
| 207 | DOK2 | 0.9944[0.8452-1.17] | 0.946 |
| 208 | SLC25A37 | 1.247[1.025-1.519] | 0.0277 |
| 209 | PLIN2 | 1.063[0.9075-1.246] | 0.448 |
| 210 | CEP78 | 0.8938[0.6611-1.208] | 0.466 |
| 211 | GSN | 1.092[0.8581-1.391] | 0.473 |
| 212 | PAXX | 0.6371[0.4809-0.8441] | 0.00168 |
| 213 | DGKZ | 0.5885[0.4189-0.8268] | 0.00224 |
| 214 | SERPING1 | 1.204[1.018-1.424] | 0.0304 |
| 215 | SERPINH1 | 1.27[1.04-1.551] | 0.0191 |
| 216 | TAGLN | 1.095[0.9457-1.269] | 0.225 |
| 217 | KLRF1 | 1.108[0.9366-1.311] | 0.232 |
| 218 | ARID5B | 1.061[0.8692-1.296] | 0.559 |
| 219 | PIP4K2A | 1.134[0.9181-1.4] | 0.243 |
| 220 | FAM177A1 | 1.269[0.9302-1.731] | 0.133 |
| 221 | DST | 1.095[0.8571-1.398] | 0.469 |
| 222 | SPARCL1 | 0.9947[0.8509-1.163] | 0.947 |
| 223 | CPB1 | 1.022[0.9849-1.06] | 0.252 |
| 224 | SAMSN1 | 1.08[0.9436-1.236] | 0.264 |
| 225 | ETS2 | 1.186[0.9373-1.5] | 0.156 |
| 226 | TAGLN2 | 1.352[1.046-1.748] | 0.0214 |
| 227 | NBL1 | 1.146[0.9349-1.405] | 0.19 |
| 228 | PTMS | 0.7533[0.5793-0.9797] | 0.0346 |
| 229 | C1R | 1.167[0.987-1.38] | 0.0708 |
| 230 | SPON2 | 1.11[0.944-1.306] | 0.206 |
| 231 | CSTB | 1.217[0.9735-1.521] | 0.0847 |
| 232 | ITGB2 | 1.111[0.964-1.279] | 0.147 |
| 233 | PRMT2 | 0.7927[0.5781-1.087] | 0.149 |
| 234 | LMNA | 1.268[0.9805-1.639] | 0.0703 |
| 235 | CTRC | 1.012[0.9723-1.053] | 0.559 |
| 236 | ATF3 | 1.114[0.9643-1.286] | 0.143 |
| 237 | DENND2D | 1.188[0.9173-1.539] | 0.191 |
| 238 | S100A11 | 1.378[1.139-1.667] | 0.00096 |
| 239 | COL6A3 | 1.185[1.042-1.348] | 0.00966 |
| 240 | FSTL1 | 1.169[0.9905-1.38] | 0.0647 |
| 241 | IGFBP7 | 1.047[0.8501-1.29] | 0.664 |
| 242 | PYHIN1 | 1.007[0.9024-1.124] | 0.898 |
| 243 | ANXA5 | 1.304[1.005-1.692] | 0.0457 |
| 244 | SAMD3 | 0.9227[0.8096-1.052] | 0.228 |
| 245 | FABP5 | 1.027[0.8418-1.254] | 0.791 |
| 246 | COL1A2 | 1.163[1.021-1.323] | 0.0226 |
| 247 | BRI3 | 1.175[0.8365-1.65] | 0.352 |
| 248 | CTSB | 1.288[1.02-1.626] | 0.0332 |
| 249 | IFI27 | 1.249[1.095-1.425] | 0.00095 |
| 250 | HTRA1 | 1.062[0.88-1.282] | 0.53 |
| 251 | PLEKHF1 | 1.132[0.9416-1.36] | 0.187 |
| 252 | B2M | 1.36[1.064-1.738] | 0.0142 |
| 253 | NNMT | 1.189[0.9893-1.428] | 0.065 |
| 254 | SLFN5 | 1.319[1.081-1.61] | 0.00649 |
| 255 | CD3D | 1.036[0.9203-1.167] | 0.557 |
| 256 | TPM4 | 1.551[1.191-2.018] | 0.00111 |
| 257 | LAIR2 | 1.063[0.9477-1.192] | 0.298 |
| 258 | FTH1 | 1.222[0.9186-1.626] | 0.168 |
| 259 | PTGDR | 0.9949[0.8617-1.149] | 0.944 |
| 260 | CX3CR1 | 0.936[0.8335-1.051] | 0.263 |
| 261 | ARF4 | 1.331[1.001-1.771] | 0.0494 |
| 262 | RAB31 | 1.221[1.038-1.436] | 0.0157 |
| 263 | COL3A1 | 1.158[1.023-1.312] | 0.0205 |
| 264 | IL7R | 1.046[0.9436-1.159] | 0.394 |
| 265 | CTRB1 | 1.017[0.9813-1.054] | 0.356 |
| 266 | PPIC | 1.294[1.025-1.633] | 0.0299 |
| 267 | CXCL8 | 1.142[1.032-1.263] | 0.0101 |
| 268 | CD52 | 1.026[0.9072-1.16] | 0.686 |
| 269 | GPR183 | 1.075[0.9513-1.214] | 0.247 |
| 270 | ZEB2 | 1.025[0.8759-1.199] | 0.76 |
| 271 | CLIC3 | 1.158[1.049-1.279] | 0.00363 |
| 272 | MT1E | 1.237[1.039-1.474] | 0.017 |
| 273 | TM4SF1 | 1.384[1.161-1.649] | 0.000291 |
| 274 | KLF13 | 1.078[0.8441-1.377] | 0.548 |
| 275 | FOS | 1.083[0.9269-1.266] | 0.314 |
| 276 | JUNB | 1.061[0.8573-1.313] | 0.587 |
| 277 | HOPX | 1.103[0.9378-1.297] | 0.237 |
| 278 | CEBPB | 1.338[1.084-1.652] | 0.00677 |
| 279 | CTSW | 1.104[0.9611-1.268] | 0.162 |
| 280 | RNF213 | 1.201[0.9447-1.527] | 0.135 |
| 281 | GK5 | 1.117[0.8488-1.471] | 0.429 |
| 282 | TBC1D10C | 0.929[0.8096-1.066] | 0.294 |
| 283 | PNLIP | 1.02[0.9873-1.054] | 0.232 |
| 284 | UCP2 | 0.8485[0.696-1.034] | 0.104 |
| 285 | A2M | 0.9881[0.8245-1.184] | 0.897 |
| 286 | NUPR1 | 1.23[1.032-1.467] | 0.0209 |
| 287 | BASP1 | 1.109[0.9405-1.308] | 0.218 |
| 288 | CAVIN1 | 1.147[0.928-1.417] | 0.205 |
| 289 | JUN | 1.084[0.8704-1.35] | 0.472 |
| 290 | PLEC | 1.258[1.048-1.509] | 0.0136 |
| 291 | MAF | 1.125[0.9178-1.38] | 0.256 |
| 292 | GIMAP7 | 0.9139[0.7942-1.052] | 0.209 |
| 293 | SYCN | 1.014[0.9758-1.054] | 0.475 |
| 294 | MYADM | 1.009[0.8129-1.252] | 0.935 |
| 295 | PRF1 | 1.069[0.9062-1.262] | 0.427 |
| 296 | S1PR5 | 1.006[0.8488-1.191] | 0.949 |
| 297 | FCRL6 | 1.012[0.8866-1.156] | 0.857 |
| 298 | C1S | 1.164[1.002-1.353] | 0.0465 |
| 299 | BGN | 1.115[0.9289-1.338] | 0.243 |
| 300 | LCK | 1.091[0.9537-1.247] | 0.205 |
| 301 | ASCL2 | 0.8305[0.7357-0.9374] | 0.00266 |
| 302 | SOCS3 | 1.057[0.8958-1.247] | 0.511 |
| 303 | PDE4B | 0.9553[0.8202-1.113] | 0.557 |
| 304 | TCEAL9 | 1.15[0.8533-1.55] | 0.358 |
| 305 | MYBL1 | 1.103[0.9027-1.348] | 0.337 |
| 306 | IKZF1 | 0.9984[0.8881-1.122] | 0.979 |
| 307 | IRS2 | 0.9361[0.7432-1.179] | 0.575 |
| 308 | BTN3A2 | 1.216[0.9908-1.493] | 0.0612 |
| 309 | ARHGAP30 | 1.002[0.8637-1.163] | 0.976 |
| 310 | GPATCH8 | 1.067[0.8098-1.405] | 0.647 |
| 311 | CMC1 | 1.062[0.7407-1.524] | 0.742 |
| 312 | MT1X | 1.211[1.002-1.464] | 0.0473 |
| 313 | ARL4C | 1.266[1.052-1.523] | 0.0127 |
| 314 | HBA2 | 0.9551[0.8675-1.052] | 0.35 |
| 315 | S100A16 | 1.414[1.161-1.722] | 0.000571 |
| 316 | S100A13 | 1.328[1.034-1.706] | 0.0264 |
| 317 | SPATS2L | 1.512[1.163-1.965] | 0.00199 |
| 318 | S100A4 | 1.152[1.028-1.292] | 0.0152 |
| 319 | CD55 | 1.163[0.9933-1.361] | 0.0606 |
| 320 | EVL | 0.8331[0.707-0.9816] | 0.0291 |
| 321 | TCF4 | 1.059[0.8838-1.268] | 0.536 |
| 322 | HSH2D | 1.261[1.044-1.522] | 0.0161 |
| 323 | CD47 | 1.444[1.034-2.016] | 0.031 |
| 324 | DTHD1 | 1.079[0.9671-1.205] | 0.173 |
| 325 | SERPINA1 | 0.9131[0.8069-1.033] | 0.15 |
| 326 | SPN | 1.002[0.8883-1.131] | 0.971 |
| 327 | GZMM | 0.9173[0.7951-1.058] | 0.237 |
| 328 | PSAP | 0.8119[0.6136-1.074] | 0.145 |
| 329 | S100A10 | 1.35[1.116-1.634] | 0.00199 |
| 330 | MBP | 0.9082[0.7178-1.149] | 0.422 |
| 331 | TPM2 | 1.079[0.9132-1.274] | 0.373 |
| 332 | SH2D1B | 1.092[0.9404-1.269] | 0.247 |
| 333 | CD247 | 0.9941[0.8682-1.138] | 0.931 |
| 334 | SELENOM | 0.8197[0.6541-1.027] | 0.0842 |
| 335 | FCGR3A | 1.122[0.9869-1.275] | 0.0789 |
| 336 | LIME1 | 0.7985[0.6839-0.9322] | 0.0044 |
| 337 | HSPA1B | 1.082[0.9215-1.271] | 0.335 |
| 338 | HSPA1A | 1.106[0.9615-1.272] | 0.159 |
| 339 | NCR3 | 0.9776[0.8625-1.108] | 0.723 |
| 340 | HLA-C | 1.24[0.9508-1.618] | 0.112 |
| 341 | HLA-E | 1.249[0.9443-1.652] | 0.119 |
| 342 | HLA-F | 1.228[0.9983-1.509] | 0.0519 |
| 343 | PRSS1 | 1.031[0.9905-1.073] | 0.136 |
| 344 | PSMB10 | 1.281[0.988-1.661] | 0.0617 |
| 345 | ADGRG1 | 1.133[0.8695-1.477] | 0.355 |
| 346 | KLRC3 | 0.9105[0.7633-1.086] | 0.297 |
| 347 | HLA-A | 1.094[0.8032-1.49] | 0.569 |
| 348 | IGKC | 1.036[0.9606-1.117] | 0.361 |
| 349 | TRBC1 | 0.9792[0.8706-1.101] | 0.726 |
| 350 | TRDC | 1.015[0.8792-1.173] | 0.835 |
| 351 | PPP1CB | 1.36[1-1.848] | 0.0498 |
| 352 | KLRK1 | 0.9114[0.7934-1.047] | 0.19 |
| 353 | CELA3B | 1.02[0.9831-1.058] | 0.293 |
| 354 | LINC00623 | 0.9959[0.7673-1.293] | 0.975 |
| 355 | C12orf75 | 1.049[0.8954-1.228] | 0.555 |
| 356 | LINC01871 | 1.087[0.9334-1.265] | 0.284 |
| 357 | APOBEC3G | 1.224[0.996-1.505] | 0.0546 |
| 358 | AMY2A | 1.014[0.9777-1.051] | 0.46 |
| 359 | NEAT1 | 1.041[0.897-1.209] | 0.594 |
| 360 | SELENOP | 0.9346[0.804-1.086] | 0.379 |
| 361 | TXNIP | 1.184[0.9725-1.442] | 0.0925 |
| 362 | MTRNR2L12 | 0.8447[0.701-1.018] | 0.0762 |
| 363 | KCNQ1OT1 | 1.015[0.8451-1.22] | 0.87 |
| 364 | CCL5 | 1.091[0.9516-1.25] | 0.212 |
| 365 | CCL4 | 0.9868[0.8605-1.132] | 0.849 |
| 366 | PRSS2 | 1.038[0.995-1.084] | 0.084 |
| 367 | CCL3L1 | 0.8964[0.7923-1.014] | 0.0827 |
| 368 | MARCKS | 1.051[0.7716-1.432] | 0.752 |
| 369 | CCL3 | 0.9437[0.8218-1.084] | 0.411 |
| 370 | TRG-AS1 | 1.031[0.8978-1.184] | 0.666 |
| 371 | C5orf566 | 1.024[0.7961-1.31] | 0.855 |

**
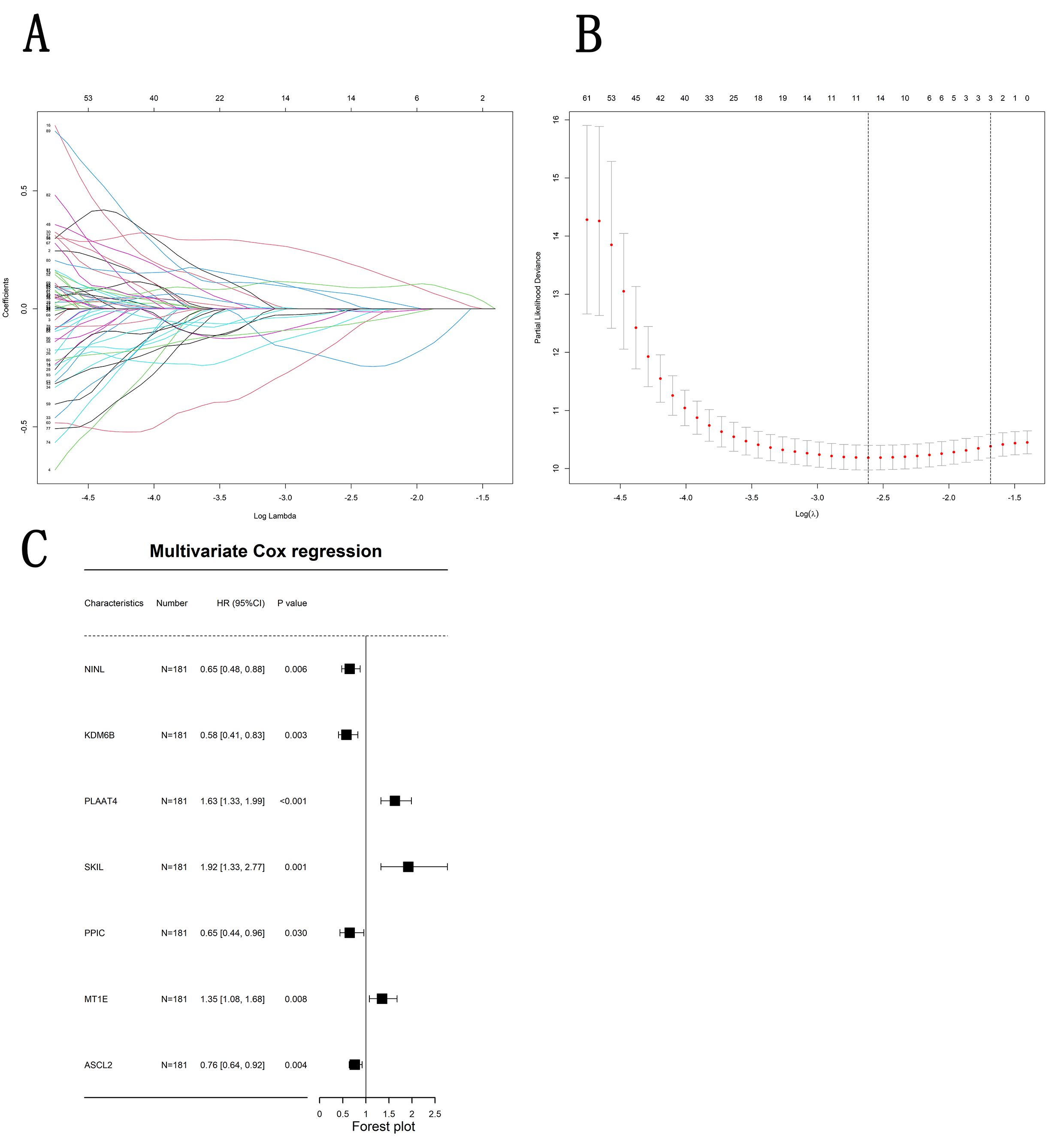
**

**Fig.S1 Construction of the NKCMGS** A: 22 representative OS-associated NK cell marker genes was screened by Lasso regression; B: Lasso regression parameters were adjusted by 100-fold cross validation; C: Multivariate Cox regression analysis of 7 prognostic genes.


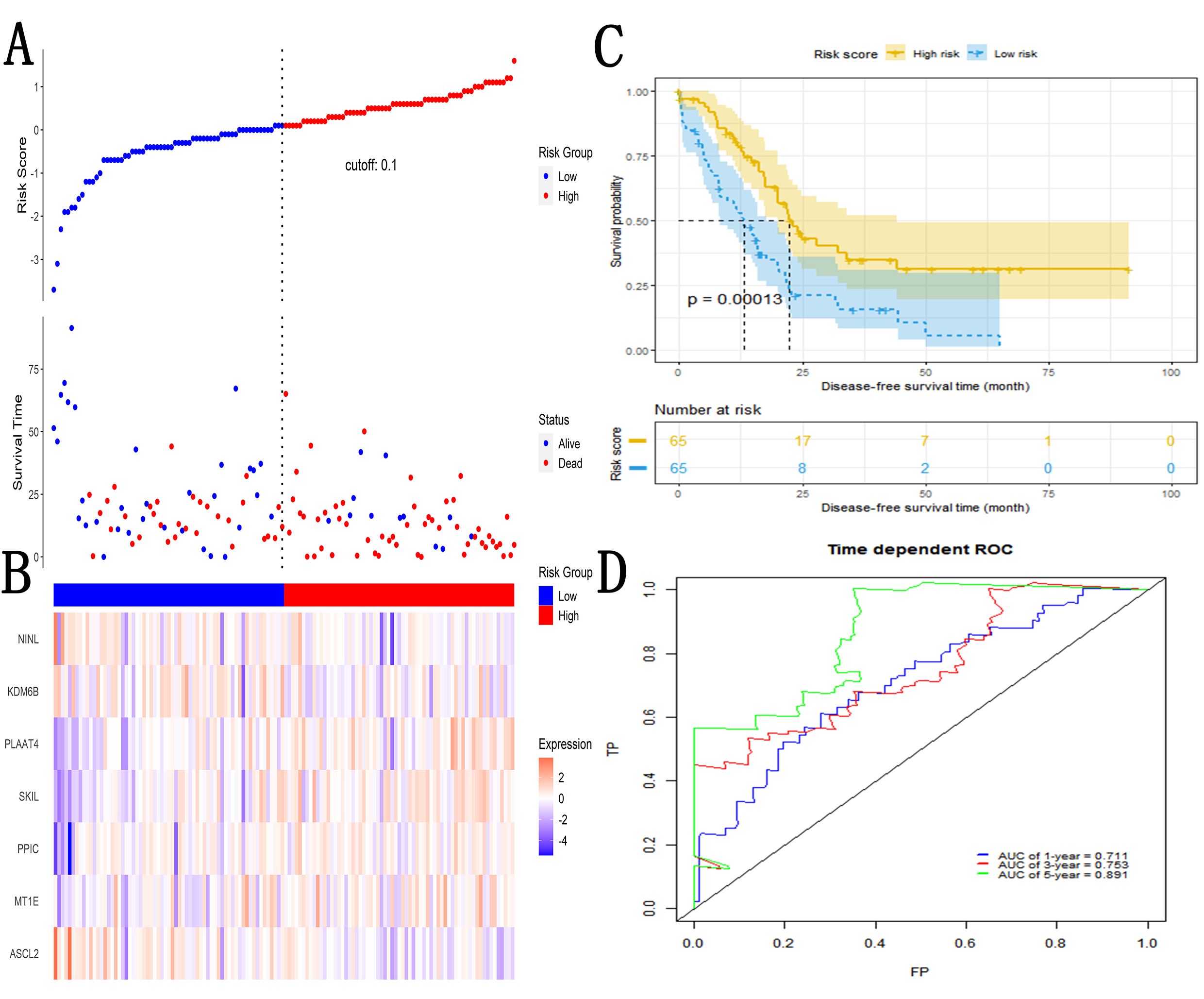


**Fig.S2 Validation of the prognostic value of NKCMGS for DFS in PDAC** A: Distribution of the vital status of PDAC patients inthe different risk groups; B: The heatmap represents the relationship between the expression of 7 NK marker genes constituting NKCMGS and vital status in PDAC patients; C: The Kaplan-Meier curve was used to compare DFS of the different risk groups; D: The ROC curve was used to evaluate the predictive ability of NKCMGS for 1-year, 3-year and 5-year DFS in PDAC patients.

**Table S3 The baseline characteristics of TCGA-PAAD datasets and 6 GSE datasets.**

| **Characteristics** | **TCGA (N=176)** | **GSE21501 (N=102)** | **GSE28735 (N=43)** | **GSE57495 (N=63)** | **GSE62452 (N=66)** | **GSE71729 (N=125)** | **GSE78229 (N=49)** |
| --- | --- | --- | --- | --- | --- | --- | --- |
| **Age** |  |  |  |  |  |  |  |
| ≤65 | 93 | / | / | / | / | / | / |
| >65 | 83 | / | / | / | / | / | / |
| **Gender** |  |  |  |  |  |  |  |
| Female | 80 | / | / | / | / | / | / |
| Male | 96 | / | / | / | / | / | / |
| **Site** |  |  |  |  |  |  |  |
| Head | 128 | / | / | / | / | / | / |
| Body and tail | 29 | / | / | / | / | / | / |
| Overlapping | 2 | / | / | / | / | / | / |
| NA | 17 | / | / | / | / | / | / |
| **T stage** |  |  |  |  |  |  |  |
| T1-T2 | 31 | 18 | / | / | / | / | / |
| T3-T4 | 143 | 80 | / | / | / | / | / |
| TX | 2 | 4 | / | / | / | / | / |
| **N stage** |  |  |  |  |  |  |  |
| N0 | 53 | 28 | / | / | / | / | / |
| N1 | 122 | 73 | / | / | / | / | / |
| NX | 1 | 1 | / | / | / | / | / |
| **TNM stage** |  |  |  |  |  |  |  |
| Ⅰ-Ⅱa | 49 | / | / | 30 | 14 | / | 14 |
| Ⅱb-Ⅳ | 124 | / | / | 33 | 52 | / | 35 |
| NA | 3 | / | / | 0 | 0 | / | 0 |
| **Grade** |  |  |  |  |  |  |  |
| G1-G2 | 124 | / | / | / | 34 | / | 26 |
| G3-G4 | 50 | / | / | / | 31 | / | 22 |
| GX | 2 | / | / | / | 1 | / | 1 |
| **Vital status** |  |  |  |  |  |  |  |
| Alive | 84 | 36 | 13 | 21 | 16 | 41 | 14 |
| Death | 92 | 66 | 29 | 42 | 50 | 85 | 35 |


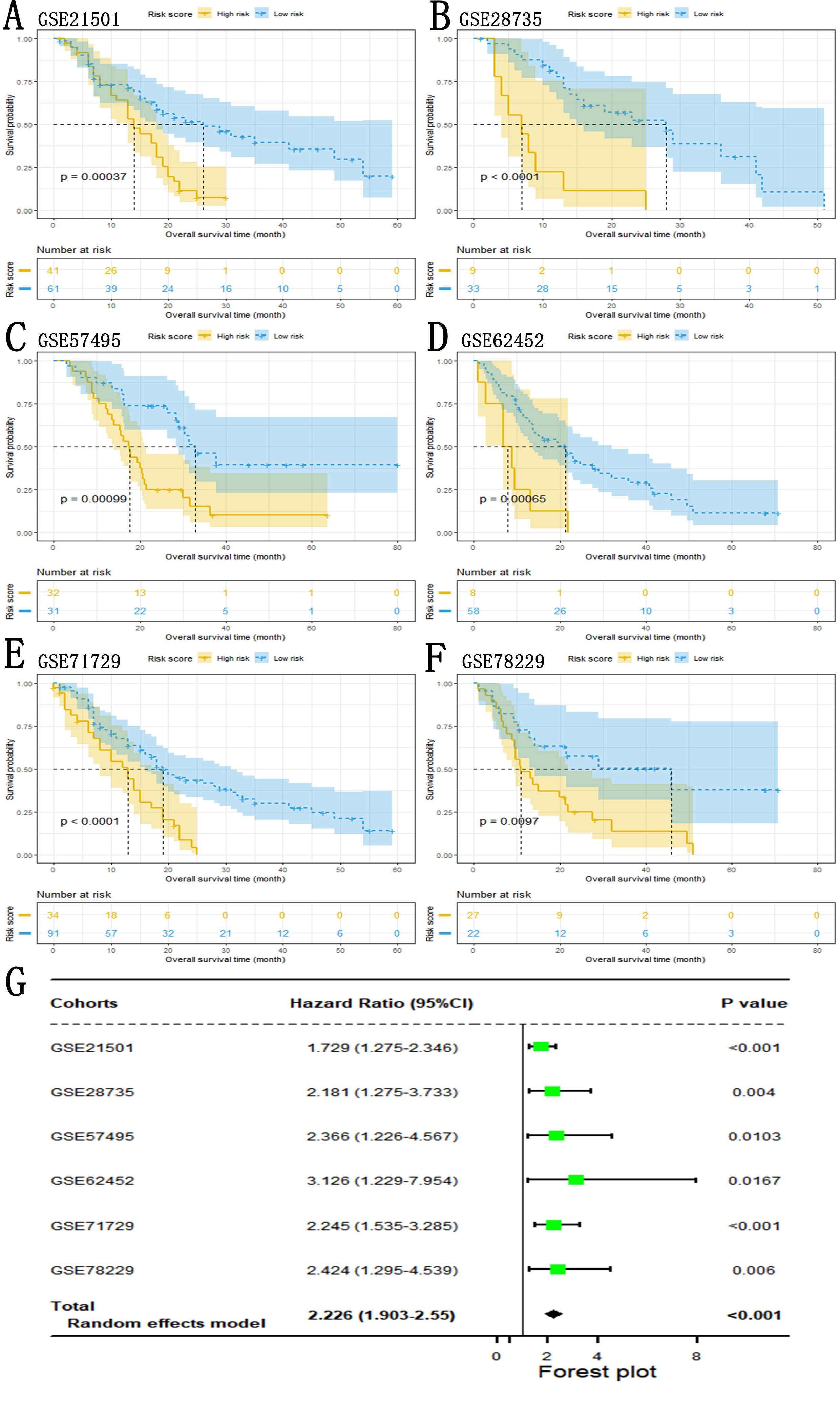


**Fig.S3 Validation of the prognostic value of NKCMGS in six independent GSE datasets** A: Kaplan-Meier curves for different risk groups in the GSE21501 dataset; B: Kaplan-Meier curves for different risk groups in the GSE28735 dataset; C: Kaplan-Meier curves for different risk groups in the GSE57495 dataset; D: Kaplan-Meier curves for different risk groups in the GSE62452 dataset; E: Kaplan-Meier curves for different risk groups in the GSE71729 dataset; F: Kaplan-Meier curves for different risk groups in the GSE78229 dataset; G: The Meta-analysis was used to calculate the synthesis effects for the six datasets.


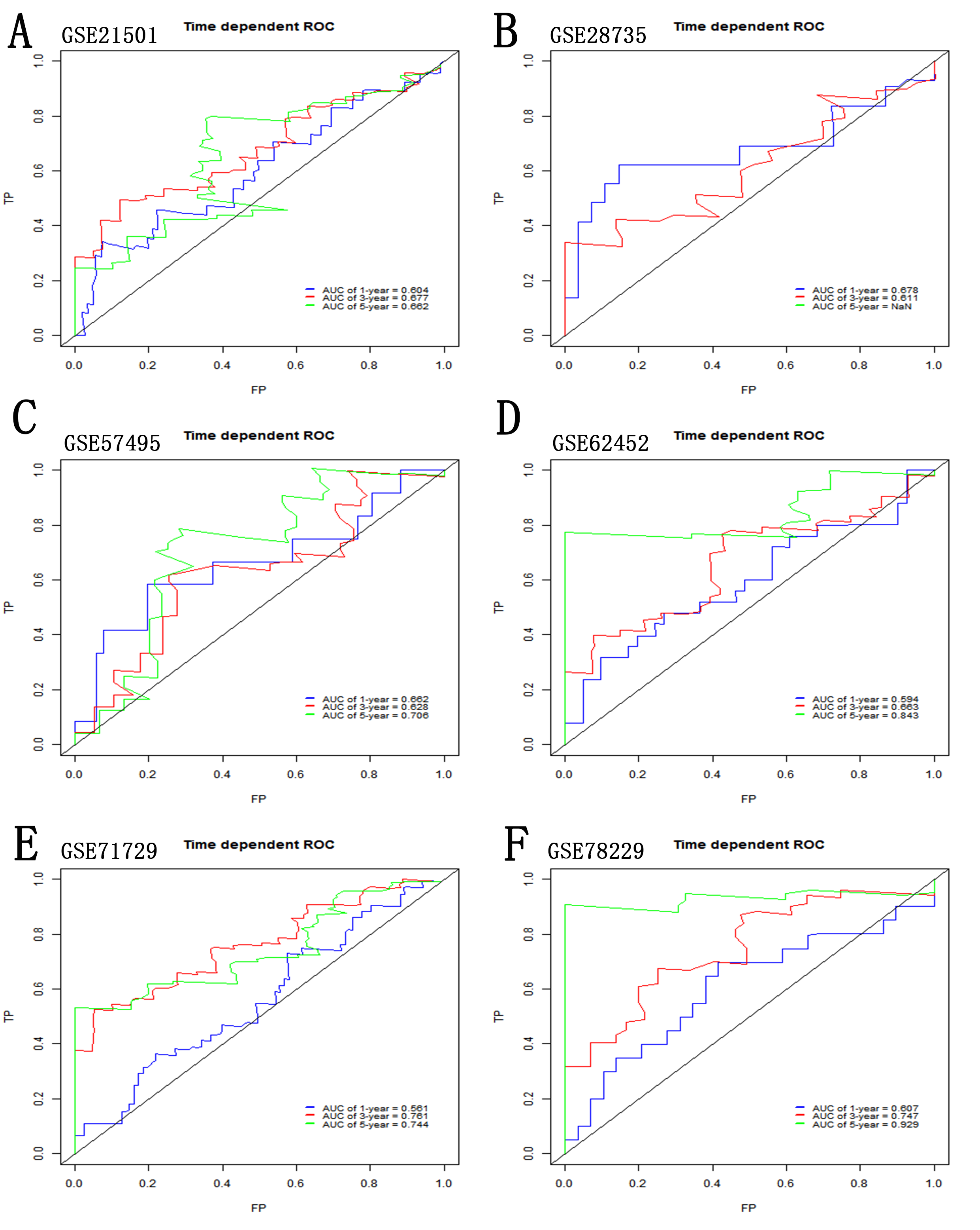


**Fig.S4 The ROC curve was used to evaluate the predictive ability of NKCMGS for 1-year, 3-year and 5-year survival rate in 6 independent GSE datasets for validation** A: ROC curves in the GSE21501 dataset; B: ROC curves in the GSE28735 dataset; C: ROC curves in the GSE57495 dataset; D: ROC curves in the GSE62452 dataset; E: ROC curves in the GSE71729 dataset; F: ROC curves in the GSE78229 dataset;


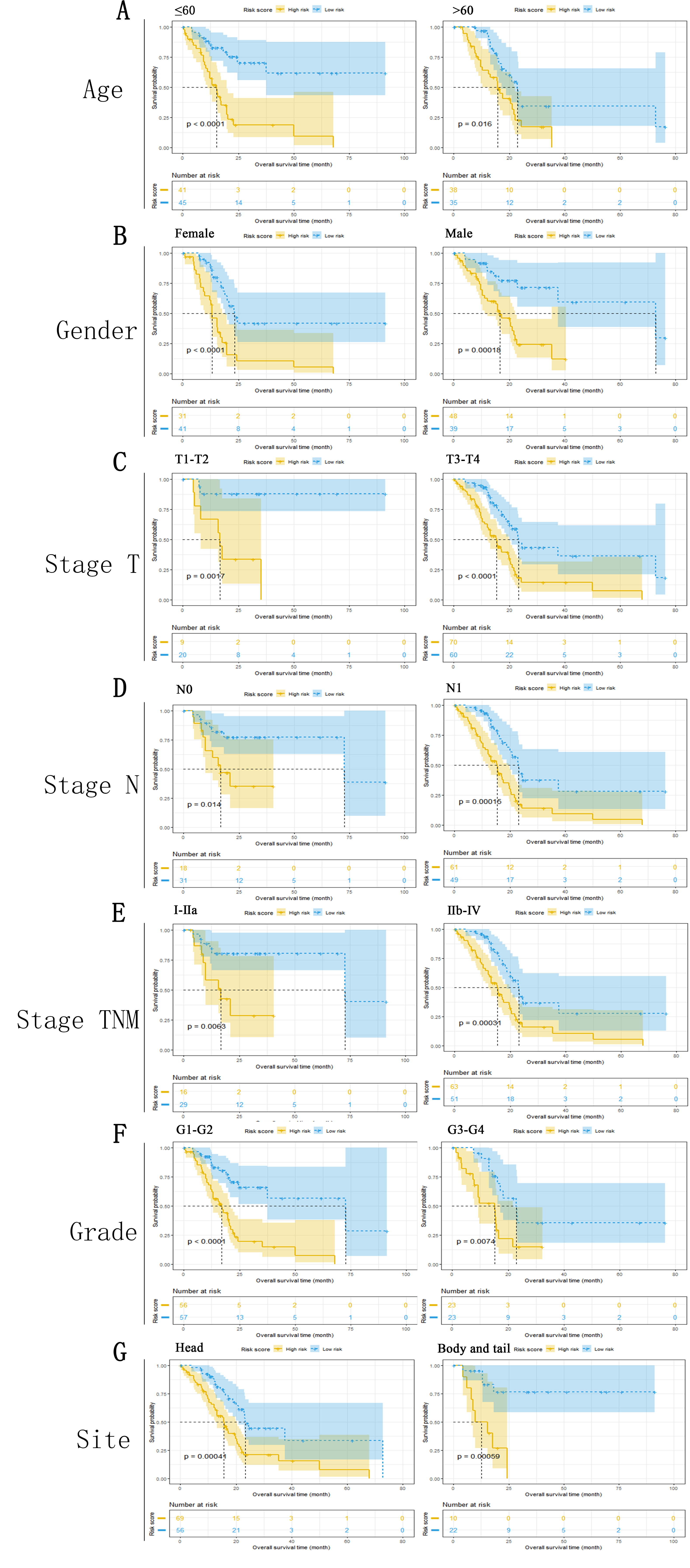


**Fig.S5 The prognostic value of NKCMGS in different clinical subgroups** A: Age (≤60 years-old/>60 years-old); B: Gender (Female/male); C: Stage T (T1-T2/T3-T4); D: Stage N (N0/N1); E: Stage TNM (Ⅰ-Ⅱa/Ⅱb-Ⅳ); F: Grade (G1-G2/G3-G4); G: Site of pancreas (Head of pancreas/body and tail of pancreas).


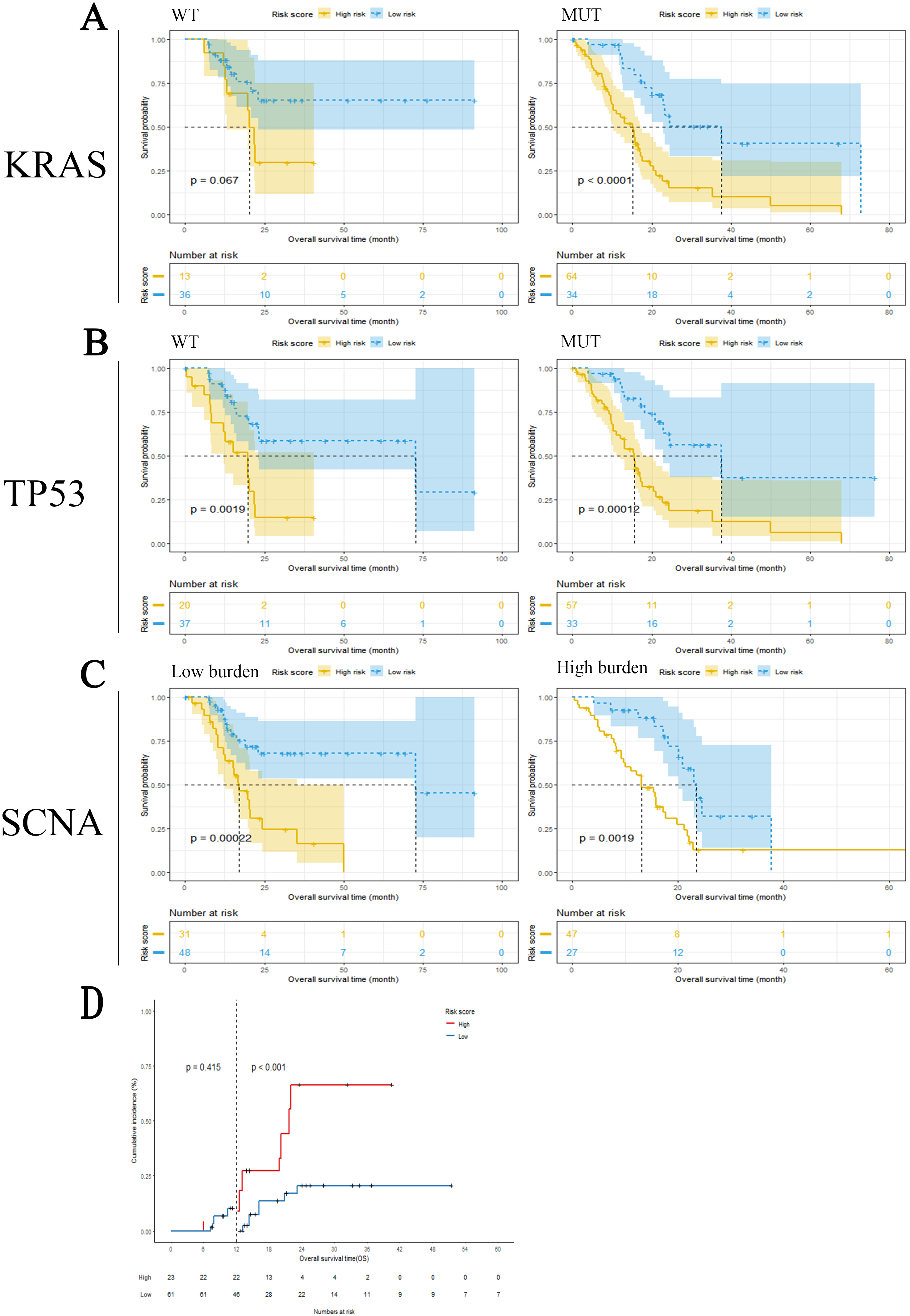


**Fig.S6 Prognostic value of NKCMGS in different mutational states** A: The Kaplan-Meier curves for different KRAS mutational states; B: The Kaplan-Meier curves for different TP53 mutational states; C: The Kaplan-Meier curves for different SCNA; D: The landmark analysis for KRAS-WT.
